# The avatar principle: exosomal dynamics guiding tumor adaptation and next-generation therapeutic strategies

**DOI:** 10.1186/s12951-026-04089-8

**Published:** 2026-02-11

**Authors:** Juan C. Baena, Sergio Camilo Cabrera-Salcedo, Yesenia Carrera Suárez, Juan M. Biancha-Vasco, Lady J. Rios-Serna, M. Daniela García-Mantilla, Manuela Estrada-Schweineberg, Juan Sebastian Victoria Hincapie, Alejandro Toro-Pedroza, Juan Esteban Garcia-Robledo, Carlos A. Cañas, Joshua Ortiz-Guzman, Alexandre Loukanov

**Affiliations:** 1 Division of Oncology, Department of Medicine, Fundación Valle del Lili, ICESI University, Cali, Colombia; 2 LiliCAR-T Group, Fundación Valle del Lili, ICESI, Cali, Colombia; 3 Faculty of Health Sciences, Universidad Icesi, Cali, Colombia; 4 Faculty of Engineering, Design and Applied Sciences, Universidad Icesi, Cali, Colombia; 5 Master’s Programme in Biopharmaceuticals, Department of Pharmacy, Uppsala University, Uppsala, Sweden; 6 Universidad Icesi, CIRAT: Centro de Investigación en Reumatología, Autoinmunidad y Medicina Traslacional, Cali, Colombia; 7 Hematology department, Fundacion Santa Fe de Bogota, Bogotá, Colombia; 8 Centro de Investigación Hemato Oncológico (CIHO), IDC Instituto de Cáncer Hemato Oncólogos, Cali, Colombia; 9 Division of Rheumatology, Department of Medicine, Fundación Valle del Lili, ICESI University, Cali, Colombia; 10 Director of Clinical Research, Rio Grande Urology, El Paso, TX USA; 11 Prodigy Cells Labs, LLC, Doral, FL USA; 12 Department of Chemistry and Materials Science, National Institute of Technology, Gunma College, Maebashi, Japan; 13 Laboratory of Engineering Nanobiotechnology, University of Mining and Geology ″St. Ivan Rilski″, Sofia, Bulgaria; 14 Center for Cell and Gene therapy, Baylor College of Medicine, Houston, TX USA

**Keywords:** Exosomes, Tumor-derived exosomes (TDEs), Extracellular vesicles (EVs), Chimeric antigen receptor (CAR) t cells, CAR-T exosomes, Immunotherapy, Tumor microenvironment (TME), Pre-metastatic niche, Exosome engineering, Nanotechnology, CRISPR/Cas9 delivery, Drug delivery, Cell-free therapy, Immune suppression, Cancer resistance

## Abstract

Exosomes are nanoscale extracellular vesicles that transfer proteins, nucleic acids, and lipids, reflecting the state of their parent cells. A persistent scientific challenge is that tumor-derived exosomes (TDEs) facilitate immune evasion, remodel the tumor microenvironment, and create premetastatic niches, intensifying tumor aggressiveness and undermining therapeutic efficacy, ultimately narrowing treatment options to palliative strategies in advanced settings. Yet their dual roles as suppressive agents and potential therapeutic tools remain poorly integrated within current cancer immunotherapy frameworks. This review examines the molecular mechanisms underlying TDE-mediated immune suppression and therapeutic resistance, while also highlighting engineering strategies to exploit or counteract exosome biology. Exosomes derived from chimeric antigen receptor (CAR) T cells preserve antigen specificity and cytotoxic components without the risks of uncontrolled proliferation or cytokine release, offering a safer class of cell free immunotherapies. Advances in genetic engineering, hybrid vesicle design, and nanotechnology have extended exosome applications to the delivery of CRISPR/Cas systems, chemotherapeutic agents, immunoregulatory RNAs, and vaccines, with liposome or nanoparticle integration enhancing targeting and efficacy. Remaining obstacles include the lack of standardized protocols, scalability issues in production, and unresolved regulatory frameworks. Drawing on The Art of War, exosomes can be envisioned as avatars of strategy, discreet messengers capable of undermining host defenses while simultaneously carrying the potential to redirect immunity against the tumor. By embodying both deception and counterattack, they illustrate the capacity to penetrate hidden barriers and redefine the therapeutic battlefield, opening new horizons for precision cancer immunotherapy.

**Graphical abstract:**

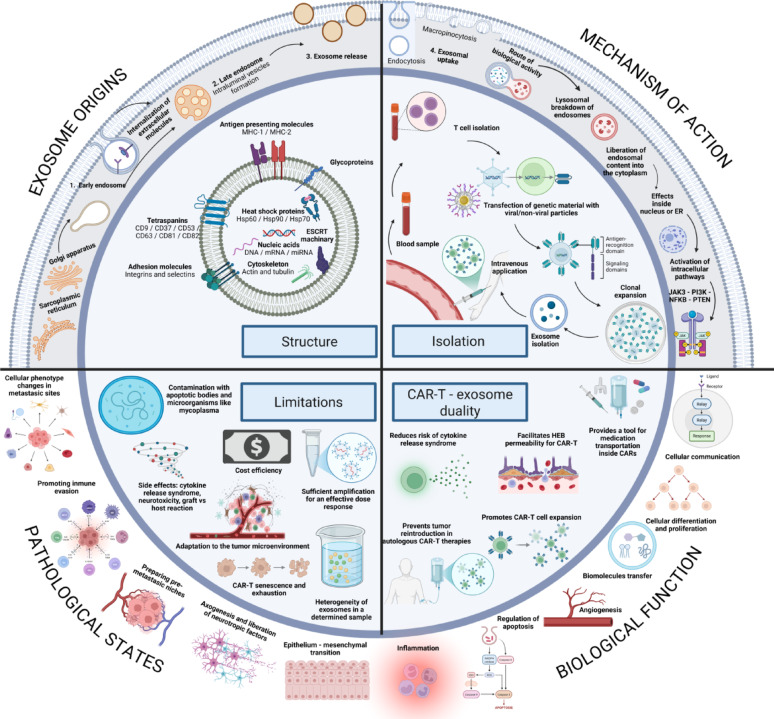

## Introduction

Exosomes are the smallest type of extracellular nanoscale vesicles (EVs) (30–150 nm) secreted by nearly all cell types and function as key mediators of intercellular communication through their ability to transport proteins, lipids, and nucleic acids between cells [1–5]. Their molecular content mirrors the physiological or pathological status of their cells of origin, enabling them to influence immune responses, gene expression, and cell behavior across diverse biological contexts [6, 7].

In cancer biology, tumor-derived exosomes (TDEs) have emerged as critical mediators of tumor immune evasion and microenvironment remodeling. These vesicles carry tumor-specific antigens, oncogenic proteins, and immunomodulatory molecules that systematically reprogram the tumor microenvironment (TME) [8, 9]. By delivering oncogenic proteins, immunosuppressive ligands, and regulatory RNAs, TDEs reprogram the TME to promote regulatory T cell expansion, polarization of tumor-associated macrophages (TAMs), suppression of cytotoxic lymphocytes, angiogenesis, extracellular matrix (ECM) remodeling, and formation of pre-metastatic niches (PMN), reflecting at a nanoscale all hallmarks of cancer [10]. These features position TDEs as both a therapeutic target and a platform, offering a potential use in cell-free immunotherapy or as delivery vehicles for precision oncology.

**Fig. 1:**
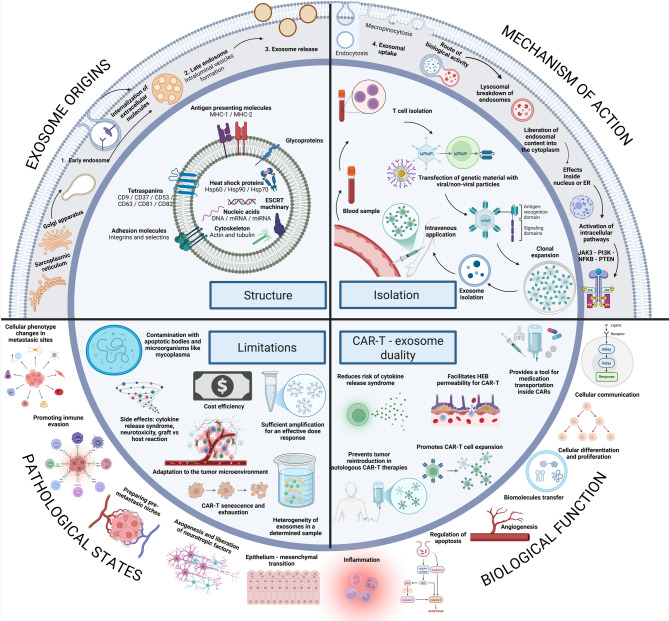
Biological and therapeutic exosomes: a general outlook at their structure, manufacture, and uses

Recent developments in chimeric antigen receptor (CAR) T-cell therapy have extended to the exosomal domain. CAR T-derived exosomes preserve key effector functions, including cytotoxic proteins and CAR surface expression, while lacking replicative capacity and immune checkpoint molecules such as programmed death-1 (PD-1). This profile reduces the risk of cytokine release syndrome (CRS) and immune exhaustion, supporting their utility in engineered immunotherapies [11]. Synthetic strategies including CRISPR/Cas9 loading and hybrid constructs are being explored to enhance their specificity and potency [12]. Nevertheless, challenges remain in standardization, large-scale manufacturing, and clinical translation. This review explores the molecular basis of TDEs-mediated immune suppression and resistance and highlights engineering approaches aimed at leveraging or counteracting exosome biology in cancer therapy (Fig. 1). Although TDEs constitute the primary pathogenic focus of this review, we also examine exosomes released by T cells, including CAR T cells, as they represent one of the most clinically advanced and experimentally tractable class of therapeutic exosomes. Within the heterogeneous TME, multiple cell types secrete vesicles that modulate tumor progression; however, T cell–derived exosomes emerge as **a** key subset currently subjected to systematic engineering, good manufacturing practice (GMP) compatible production, and early clinical evaluation. Their inclusion provides a mechanistic and translational counterpoint to TDE-mediated immune suppression and therapeutic resistance, illustrating how exosomal pathways co-opted by tumors can be redirected to achieve antitumor efficacy.

In this review, we define the “Avatar Principle” as the mechanistic notion that exosomes act as molecular surrogates of their cells of origin, faithfully transmitting their genomic, proteomic, lipidomic, and regulatory signatures. Through precisely regulated ESCRT-dependent and ceramide-mediated pathways, together with RNA-binding protein–guided sorting, exosomes encapsulate oncogenic drivers, immunomodulatory ligands, metabolic regulators, and non-coding RNAs that reproduce the functional phenotype and adaptive programs of the parental tumor cell. In this way, exosomes work as operational extensions of malignant cells nanoscale agents capable of executing immune suppression, stromal reprogramming, therapeutic resistance, and pre-metastatic niche conditioning across spatial distances.

Within this architecture, we used the strategic elements borrowed from The Art of War that serve not as literary embellishment but as an interpretive framework that parallels established biological evidence. Sun Tzu’s principle that the outcome of conflict is shaped long before direct confrontation resonates with the documented capacity of TDEs to precondition the microenvironment: they reorganize the extracellular matrix, manipulate cellular signaling networks, alter metabolic gradients, and modulate immune surveillance, thereby shaping the biological “terrain” to the tumor’s advantage. Similarly, their ability to disseminate regulatory information via miRNAs, surface ligands such as PD-L1, and stress-response proteins reflects a strategic distortion of host communication pathways, akin to controlling the flow of intelligence in warfare [13].

Accordingly, this review is structured into three major sections: (i) the molecular and immunological mechanisms by which TDEs promote tumor progression; (ii) strategies designed to halt exosomal spread, disrupt TDE biogenesis, or counteract their immunosuppressive functions; and (iii) engineering approaches that repurpose exosomes including CAR-T-derived vesicles and hybrid nanoplatforms as therapeutic carriers capable of overcoming current barriers in cancer treatment. This crucial perspective not only provides conceptual coherence but also frames exosomes as both agents of deception and instruments of counterattack within the evolving landscape of cancer therapy.

Exosomes are nanoscale EVs (30–200 nm) generated via the endosomal pathway through intraluminal vesicle formation within multivesicular bodies and subsequent fusion with the plasma membrane. They interact with recipient cells through receptor–ligand binding, membrane fusion, or endocytosis, delivering bioactive cargo that modulates intracellular signaling, gene expression, immune responses, angiogenesis, proliferation, and apoptosis. Structurally, exosomes are composed of a lipid bilayer enriched in tetraspanins (CD9, CD63, CD81), MHC molecules, adhesion proteins, heat shock proteins, and diverse nucleic acids and cytoskeletal components. TDEs promote immune evasion, therapy resistance, and premetastatic niche formation by reprogramming immune cells, whereas CAR-T–derived exosomes enhance targeted cytotoxicity, immune activation, and drug delivery while reducing CRS and improving central nervous system penetration. Despite their therapeutic promise, clinical translation is limited by challenges in large-scale production, vesicle heterogeneity, CAR-T exhaustion, TME adaptation, purification standardization, cost-effectiveness, and potential toxicities including neurotoxicity and GvHD.

## Know your enemy, know yourself: Understanding exosome biology in health and disease

The traditional classification of EVs into exosomes (30–150 nm), microvesicles (100–1000 nm), and apoptotic bodies (> 1 μm) remains useful, but recent work demonstrates substantial heterogeneity and overlap among these populations. Proteomic analyses show that EV identity is more accurately defined by biogenesis pathway, physical properties, molecular composition, and context dependent markers rather than size alone. The International Society for Extracellular Vesicles recommends the use of the general term extracellular vesicles with explicit reporting of size, density, biochemical markers, and, when possible, biogenetic origin. The MISEV2023 guidelines further advise against relying on size based categorization and emphasize transparent reporting of isolation procedures, marker selection, and functional assays to delineate EV subtypes. In addition, emerging nanoparticle classes such as exomeres and supermeres illustrate the complexity of the extracellular nanoparticle spectrum and reinforce the need for precise terminology and rigorous methodology ​ [2, 14–18]​.​ Exosomes are formed via inward budding of endosomal membranes, resulting in intraluminal vesicles (ILVs) that are secreted upon fusion of multivesicular bodies (MVBs) with the plasma membrane [19, 20]. Microvesicles originate through outward budding of the plasma membrane, while apoptotic bodies are released during the late stages of programmed cell death [21, 22].

In the human body, EVs contribute to essential physiological activities, such as metabolic regulation, gene expression, and immune signaling. However, they have also been linked to the development and progression of several diseases, including cancer, diabetes, autoimmune disorders, and neurodegenerative conditions [2, 23–26]. Their uptake by recipient cells occurs via receptor-ligand binding, direct membrane fusion, or endocytic pathways [21, 27]. Distribution and biological fate are influenced by physicochemical parameters such as size, surface composition, and environmental pH [28–30]​.

The cargo of exosomes includes transmembrane and cytosolic proteins such as tetraspanins, heat shock proteins, integrins as well as mRNAs, microRNAs, DNA fragments, and lipid species (Table 1). This composition determines their tropism, immunomodulatory capacity, and potential for therapeutic engineering. Exosomes circulate in biological fluids including blood, cerebrospinal fluid, and urine, and can cross barriers such as the blood brain barrier (BBB), supporting their application as non-invasive diagnostic tools [31]​. Compared to synthetic platforms like liposomes, exosomes exhibit superior biocompatibility, lower immunogenicity, and intrinsic targeting capability, reinforcing their relevance in both regenerative medicine and immuno-oncology [32, 33].

**Table 1:** Exosome molecular inventory

Cargo type	Components	Function
Membrane and membrane-associated proteins	Tetraspanins (CD81, CD63, CD9, CD82, CD37); integrins; ICAM-1; MHC-II; syndecan; flotillin 1/2; IL-6R; EGFR; TCR; CAR; GPCR; PD-L1; TGF-β; ADAM proteases	Intrinsic enzyme-linked receptors or catalytic activity; immune signaling; immune evasion (PD-L1); cell–cell interaction
	Lipid bilayer: Phosphatidylserine, phosphatidylcholine, sphingomyelin, ceramides, and cholesterol.	Enables direct fusion with target cells, Lipid-mediated signaling, protects exosomal cargo from enzymatic degradation and hostile extracellular conditions.
Anchored or post-translationally modified proteins	GPI-anchored proteins (proteoglycans, glypican-1, DAF, MAC-IP); prenylated small GTPases; BASP-1; Src; ubiquitinated, SUMOylated, or phosphorylated proteins)	Regulation of exosome biogenesis; intracellular signaling; vesicular trafficking
Biogenesis-related proteins and chaperones	ESCRT-associated proteins: ALIX, TSG101, syntenin; chaperones: HSP70, HSP90, HSP20	Exosome biogenesis; protein folding and stability
RNA	mRNA fragments (≤ 1 kb), miRNAs, snRNAs, tRNA fragments, snoRNAs, mtRNAs, piRNAs, vtRNAs, Y RNAs, circRNAs, rRNA fragments, lncRNAs	Intercellular communication; disease biomarkers; gene regulation
DNA	Genomic dsDNA; ssDNA; mtDNA; viral DNA; histone-associated DNA	Potential cytoprotective role (removal of damaged DNA); liquid biopsy biomarkers; reflection of genomic instability

### An army is forged in its barracks before marching to the battlefield: exosome biogenesis and structure

Exosomes can be generated through at least two pathways. The endocytic pathway is the most extensively characterized route of vesicular trafficking, primarily responsible for directing internalized cargo to lysosomal degradation but also capable of generating exosomes. Their formation involves the following steps: (a) invagination of the plasma membrane into an early secretory endosome; (b) formation of ILVs, also known as MVBs, through membrane budding driven by the incorporation of cargo into endosomes; (c) maturation of endosomes via acidification; and (d) extracellular release of ILVs as exosomes following fusion with the plasma membrane [34]. The Endosomal Sorting Complex Required for Transport (ESCRT) machinery orchestrates ILVs and MVBs biogenesis through four core complexes (ESCRT-0, -I, -II, and -III) and accessory proteins including ALIX and VPS4. ESCRT-0 recognizes ubiquitinated cargo, ESCRT-I and -II induce membrane deformation and cargo sequestration, and ESCRT-III drives vesicle scission followed by VPS4-mediated disassembly, with disruption of these components altering exosome size, number, and cargo. Additional regulation is provided by the syndecan–syntenin–ALIX axis, stress-responsive ESCRT-III proteins, and GPR143-mediated recruitment of HRS, which modulates exosomal proteomic composition and integrin-enriched vesicle secretion [5, 35, 36]​.

Exosome biogenesis can also proceed through ESCRT-independent, lipid-driven mechanisms dominated by ceramide generated by neutral sphingomyelinase 2, which promotes intraluminal vesicle budding and cargo sorting independently of ESCRT components. This pathway is supported by microtubule-associated protein 1 A/1B-light chain 3 and RAB31, enhancing exosome secretion and epidermal growth factor receptor enrichment, particularly in cancer cells. While both ESCRT-dependent and ceramide-mediated pathways converge on exosome release, they differ in cargo specificity: ESCRT preferentially sorts ubiquitinated membrane and immune-regulatory proteins, whereas lipid-driven mechanisms enrich tetraspanins, proteolipid protein, and lipid-associated components. Together with RNA-binding protein–mediated RNA loading, this molecular divergence shapes exosomal composition, intercellular signaling, stress adaptation, and disease progression [36].

Exosomal cargo is determined by the lineage and activation state of the parent cell, while structural variation reflects gene expression and cellular topology [28]. In this sense, exosomes can be viewed as molecular avatars of their tumors, faithfully carrying and projecting the biological identity and programmed functions. At early stages of biogenesis, exosomal diversity is shaped by both intracellular pathways and systemic factors. Dietary lipids modify vesicle membrane composition, circadian rhythm regulates secretion timing, and hormonal signals such as estrogen influence microRNA cargo. Infections increase the release of immunomodulatory vesicles, while physical activity enhances regenerative signaling [3, 37].

This diversity has functional consequences. On the one hand, TDEs carry oncogenic and immunosuppressive molecules that facilitate immune evasion and disease progression. On the other hand, exosomes released by effector T cells display immune activating properties; for example, CD8⁺ T cell-derived exosomes contain perforins, granzymes, co-stimulatory receptors, and surface proteins such as CD3, CD8, and Fas ligand (FasL) [38]. These exosomes enhance low-affinity cytotoxic T cell responses, modulate PD-1/PD-L1 interactions, and contribute to anti-tumor immunity [39]. They have been implicated in treatment response, with uPAR-positive CD8 + exosomes serving as potential biomarkers in melanoma [40]. Also, exosomes from CD4⁺ T cells, particularly regulatory T cells (Tregs), mediate immune suppression [41, 42] and express CD25, cytotoxic T-lymphocyte-associated protein 4 (CTLA-4), and CD73, and support B-cell activation, M2 macrophage polarization, and CD8⁺ T cell inhibition [43]. Their release is modulated by Rab GTPases, particularly Rab27, and they frequently carry immunoregulatory microRNAs such as miR-150-5p and miR-142-3p, which may serve as non-invasive biomarkers in immunotherapy settings [44–47].

### Within disorder, seek harmony: identifying the features of exosomes

Characterization of exosomes primarily focuses on the identification of molecular markers that distinguish exosomes subtypes and reflect their cellular origins. Tetraspanins such as CD9, CD63, and CD81 are widely recognized as exosome markers due to their involvement in the endosomal biogenesis pathway [22, 48]​. In contrast, proteins like annexin A1, annexin A2, ARRDC1, and α-actinin 4 are more specifically associated with microvesicle formation​ [21, 22, 49]. Certain molecules, including TSG101, syntenin-1, ALIX, ARF6, and VPS4, contribute to both exosome and microvesicle biogenesis, making them shared markers [22].

However, the small size of extracellular vesicles contributes to substantial variability in their membrane marker composition. For example, a 50 nm vesicle is estimated to accommodate only about 600 surface proteins, inherently limiting the range of detectable exosomal markers. Even exosomes originating from the same cell type can differ in marker presence and spatial distribution [50]. As a result, not all small EVs, often classified as exosomes, necessarily express classical tetraspanins, posing challenges for their consistent identification. To overcome these constraints, recent methodological advances have focused on single-vesicle and single-cell EV analysis. High-sensitivity flow cytometry, super-resolution and total internal reflection fluorescence microscopy, droplet-based digital immunoassays, Raman-based platforms, and microfluidic single-vesicle assays now enable multiplexed phenotyping of thousands of individual vesicles and, in some setups, the vesicle secretion profiles of single cells. These approaches begin to bridge the gap between physical characterization and functional heterogeneity, revealing EV subtypes that cannot be distinguished by classical bulk methods. Notably, Guo et al. employed a proximity barcoding assay for single-exosome proteomic profiling of plasma from colorectal cancer patients, identifying ITGB3⁺ and ITGAM⁺ exosome subpopulations with opposing effects on tumor progression and promising diagnostic and therapeutic value [51]. This type of single-exosome profiling exemplifies how methodological innovation can refine our understanding of exosome identity beyond conventional marker panels.

Isolation methods further shape how exosomes are defined and studied. Ultracentrifugation remains the most widely used technique, involving sequential centrifugation steps to eliminate cells, debris, and larger vesicles, followed by high-speed spins for exosome recovery [52]​. Density media like sucrose or iodixanol gradients are often used to enhance purity, with iodixanol being superior for distinguishing exosomes from retroviral particles [53]​. Polymer-based precipitation with polyethylene glycol (PEG) offers a convenient and scalable alternative, especially for large volumes, and was initially developed for virus isolation [54]​. Size-based methods, including ultrafiltration and size-exclusion chromatography (SEC), separate vesicles according to molecular weight or hydrodynamic diameter [55, 56]​, while immunoaffinity capture utilizes antibodies or ligands specific to exosomal surface proteins, enabling selective isolation from smaller sample volumes [57]. Commercial kits and microfluidic platforms have also emerged as rapid and user-friendly solutions for exosome recovery.

Despite this broad toolkit, no single isolation strategy simultaneously guarantees high purity, yield, and preservation of vesicle integrity. Differential ultracentrifugation and PEG precipitation can co-isolate protein aggregates, lipoproteins, and other nanoparticles; SEC may dilute samples and enrich only specific size fractions; and immunoaffinity capture is intrinsically biased toward vesicles bearing known markers, under-representing poorly characterized subpopulations [58]. These methodological trade-offs complicate cross-study comparisons and can profoundly influence the apparent molecular composition and bioactivity of “exosome” preparations, underscoring the need to interpret functional data in light of the underlying isolation protocol.

Once isolated, exosomes must be characterized to confirm their identity, morphology, and bioactive content. Structural features can be visualized through electron microscopy techniques such as TEM, SEM, or Cryo-EM, the latter preserving native architecture and avoiding fixation-related artifacts [59]​. Dynamic light scattering (DLS) and nanoparticle tracking analysis (NTA) provide information on hydrodynamic size distribution and concentration, with NTA enabling recovery of samples post-analysis [59, 60]​. Protein-based methods, including Western blot, ELISA, and flow cytometry, are employed to assess specific surface and cargo proteins [61, 62]​. For storage, protocols such as cryopreservation at –80 °C, lyophilization, or spray drying help preserve exosomal integrity depending on downstream applications [49, 58, 63]. According to International Society for Extracellular Vesicles (ISEV) recommendations, vesicles should be maintained in PBS at −80 °C for long-term preservation, avoiding freeze–thaw cycles to prevent structural degradation [7, 64, 65]. Exosomes retain functional activity for several months under these conditions, and lyophilization offers the additional advantage of enabling stable room-temperature storage [66].

## The enemy’s stratagems: Tumor-derived exosomes as instruments of deception

TDEs are secreted by malignant cells into the extracellular space. Although structurally like other EVs, TDEs are distinguished by their enrichment in tumor-specific proteins, lipids, glycans, and nucleic acids [67]. These components reflect the genomic, transcriptomic and proteomic profile of the tumor of origin and confer potent immunomodulatory properties. TDEs suppress anti-tumor immunity, modulate stromal cell function, and contribute to the formation of PMNs by reprogramming the phenotype and behavior of immune and non-immune cells​ [68]. Some examples are shown in Table 2.

**Table 2:** Examples of TDEs promoting cell proliferation across cancer types

Neoplasm	Role	Reference
Glioblastoma	TDEs from glioblastomas have been shown to induce proliferation of the human glioblastoma cell line U87.	[6]
Chronic myeloid leukemia (CML)	TDEs from CML cells stimulate autocrine cell proliferation.	[69]
Gastric cancer	TDEs from gastric cancer cells stimulate autocrine proliferation.	[70]
Bladder cancer	TDEs induce cell proliferation via activation of the Akt and ERK pathways.	[71]
Melanoma	TDEs promote in vivo tumor growth and inhibit apoptosis in murine melanoma models	[72]
Prostate cancer	TDEs from hypoxic prostate cancer cells increase invasion and motility in human prostate cancer cells	[73]

### The finest siege begins inside: how exosomes remodel the tumor microenvironment

The TME consists of blood vessels, ECM, fibroblasts, bone marrow–derived inflammatory cells, signaling molecules, and diverse immune cell populations [74]. Fibroblasts, endothelial cells, and infiltrating immune cells, the predominant stromal components, can interact with tumor cells through exosome-mediated signaling [75, 76].

TDEs suppress antitumor immunity through multiple mechanisms. In the case of CD8⁺ T lymphocytes, TDEs deliver inhibitory ligands that induce mitochondrial dysfunction, reduce expression of survival proteins including Bcl-2, Bcl-xL and cFLIP, and promote apoptosis [77]. In nasopharyngeal carcinoma, TDEs present PD-L1 to CD8⁺ T cells, reducing cytokine secretion, proliferation, and cytotoxicity, while in breast cancer, transforming growth factor-β (TGF-β) enhances PD-L1 loading, thereby suppressing T cell receptor (TCR) signaling [78]. In dendritic cells (DCs), TDEs downregulate antigen-processing machinery, major histocompatibility complex (MHC) class I and II molecules, and co-ligands receptors, resulting in defective maturation [79].

Exosomal microRNAs (miRNAs) and oncogenic proteins further induce DC anergy, impairing T and B lymphocyte activation and reducing natural killer (NK) cell cytotoxicity [80]; for example, exosomes carrying EGFR exon 19 deletion from Lewis lung carcinoma cells induce DC anergy [81]. In NK cells, TDEs decrease expression of the activating receptor NKG2D and reduce cytotoxicity, while bladder cancer exosomes enriched in miR-221-5p and miR-186-5p suppress perforin, granzyme B, and activating receptors [82]. In B lymphocytes, TDEs activate the adenosinergic pathway, increasing adenosine production, and inhibiting proliferation [83]. Within the TME, exosomal miR-1468-5p activates JAK2/STAT3 signaling in lymphatic endothelial cells by suppressing the SOCS1 promoter, thereby promoting immune tolerance [84].

TAMs, derived from bone marrow monocytes, infiltrate most tumors and undergo exosome-driven polarization toward either proinflammatory M1 or immunosuppressive, proangiogenic M2 phenotypes, characterized by anti-inflammatory cytokine secretion and ECM remodeling and induction of epithelial-to-mesenchymal transition (EMT) through TGF-beta secretion​ [85, 86]. Exosomal miRNAs contribute to this polarization through post-transcriptional regulation of target genes and activation of transcription factors such as peroxisome proliferator–activated receptor (PPAR), STAT3, and STAT6 [87]. Colorectal cancer–derived EMT exosomes carrying miR-106b downregulate programmed cell death protein 4 (PDCD4) and activate the PI3K/AKT mTOR pathway, favoring M2 differentiation [88]. Small cell lung cancer exosomes promote M2 conversion via NOD-like receptor family pyrin domain containing 6 (NLRP6)/NF-κB signaling [89]. Under hypoxic conditions, TDEs are enriched in immunomodulatory proteins and chemokines, which recruit macrophages and reinforce M2 polarization through oxidative phosphorylation and let-7a–mediated inhibition of insulin/AKT/mTOR signaling [90].

In parallel, TDEs expand Tregs and myeloid-derived suppressor cells (MDSCs), enhancing their suppressive functions through cytokine-mediated signaling and adenosine accumulation [91, 92]. TDEs also maintain CSC pluripotency and tumor heterogeneity by transferring stemness-associated signals [93]. Elevated TDEs concentrations in patient-derived fluids correlate with CD8⁺ T cell depletion, attenuated immunotherapy response, and disease progression, highlighting their relevance as prognostic and pharmacodynamic biomarkers [94].

### Let distance be illusion, nearness your truth: exosomes in pre-metastatic niche formation

TDEs promote PMN formation through immune modulation, vascular remodeling, lymphangiogenesis, and organ-specific dissemination [95]. At the immune level, TDEs secrete miR-21 and miR-29a that bind to Toll-like receptor 7 (TLR7) in mice and Toll-like receptor 8 (TLR8) in humans, triggering proinflammatory cascades that facilitate metastasis [96]. In breast cancer, exosomal miR-200b-3p is internalized by alveolar epithelial type II cells, inducing CCL2, S100A8/9, MMP9, and CSF1, recruiting MDSCs and generating an inflammatory PMN [97].

To promote colonization, TDEs remodel vasculature by suppressing endothelial tight junction proteins, including zonula occludens-1 (ZO-1), occludin, and claudin-5. Glioblastoma stem-like cells exosomes enriched in vascular endothelial growth factor A (VEGF-A) enhance angiogenesis and permeability in brain endothelial cells (ECs) [98], while hepatocellular carcinoma (HCC) exosomal miRNAs (miR-638, miR-663a, miR-3648, miR-4258) downregulate ZO-1 and VE-cadherin, increasing intrahepatic permeability [99]. Melanoma exosomes containing urokinase plasminogen activator receptor (uPAR) are internalized by ECs, upregulating VE-cadherin, EGFR, and uPAR expression in ECs, thereby amplifying pro-angiogenic signaling [100]. Hypoxia intensifies these effects: In multiple myeloma, hypoxia-induced exosomal miR-135b targets hypoxia-inducible factor 1 (HIF-1) in ECs [101], while in lung cancer, exosomal miR-23a suppresses prolyl hydroxylases (PHD1/2), stabilizing HIF-1α and driving angiogenesis [102]. In the lymphatic niche, tumor-derived exosomes drive nodal metastasis by delivering oncogenic RNAs and chemokine receptors that activate ERK/AKT signaling, disrupt immune barriers, and promote lymphatic endothelial migration, tube formation, and invasion [103–106].

TDEs also dictate organotropism: colorectal cancer (CRC) commonly spreads to the liver and lung, whereas breast cancer preferentially metastasizes to the lung, bone, and brain. Exosomal α6β4 and α6β1 integrins promote lung metastasis, while αvβ5 integrin is associated with liver metastasis [107]. Clinically, circulating exosomal integrin β3 in lung cancer patients with brain metastases correlated with survival and intracranial control after whole-brain radiotherapy [108]. In gastric cancer, exosomal EGFR is transferred to liver stromal cells, activating the hepatocyte growth factor (HGF)/c-MET axis in cancer cells and fostering liver colonization [109]. In pancreatic ductal adenocarcinoma (PDAC), exosomal macrophage migration inhibitory factor (MIF) is internalized by Kupffer cells, initiating PMN formation and liver metastasis [110]. In clear cell renal cell carcinoma (ccRCC), CD103⁺ CSCs–derived exosomes drive lung-specific metastasis [111].

Importantly, in vivo models corroborate these molecular findings by defining the temporal dynamics of PMN formation. In breast cancer models, pulmonary PMN formation begins within two weeks of tumor inoculation and is established by week four; this process can be reproduced by intravenous exosome administration [112]. In pancreatic cancer, hepatic PMN forms following retro-orbital injection of TDEs every 48 h for three weeks, marked by fibronectin deposition and F4/80⁺ macrophage infiltration. In spontaneous pancreatic cancer models, Kupffer cell activation and TGF-β upregulation occur during pancreatic intraepithelial neoplasia stages, four to six weeks prior to malignant transformation [110]. Collectively, TDEs act as systemic vectors that reprogram distant tissues, integrating immune suppression, vascular and lymphatic remodeling, and integrin-mediated tropism to establish permissive PMNs.

## Cut the flow, taint the source, and the spirit breaks: conventional methods to halt Exosomal spread

Targeting TDEs represents a promising approach to overcome immunosuppression and therapeutic resistance in cancer [113]. Effective strategies focus on disrupting exosome biogenesis, trafficking, and secretion through both ESCRT-dependent and ESCRT-independent pathways. In addition, bispecific antibodies such as CD73xEpCAM selectively block CD73-mediated adenosine production in exosomes from EpCAM-positive tumors, thereby restoring T cell function [114]. Some molecular strategies are shown in Table 3.

**Table 3:** Pharmacologic and Molecular Strategies to inhibit TDEs. The targets involve multiple molecular pathways. The ESCRT mediates intraluminal vesicle formation within multivesicular bodies, while the syndecan–syntenin–ALIX axis provides an accessory route linking membrane proteoglycans to ESCRT machinery. Lipid handling is regulated by ABC transporters, including the ABCA3 subtype, which is essential for exosome export. Vacuolar H⁺-ATPase (V-ATPase) maintains acidic conditions required for vesicle release, and EGFR signaling enhances exosome biogenesis through oncogenic pathways. Additional regulators include farnesyltransferase, which anchors vesicular trafficking proteins to membranes; cholesterol synthesis, which sustains membrane curvature and rigidity; and syntenin, an adaptor protein that stabilizes protein– protein interactions during vesiclePharmacologic and Molecular Strategies to inhibit TDEs. The targets involve multiple molecular pathways. The ESCRT mediates intraluminal vesicle formation within multivesicular bodies, while the syndecan–syntenin–ALIX axis provides an accessory route linking membrane proteoglycans to ESCRT machinery. Lipid handling is regulated by ABC transporters, including the ABCA3 subtype, which is essential for exosome export. Vacuolar H⁺-ATPase (V-ATPase) maintains acidic conditions required for vesicle release, and EGFR signaling enhances exosome biogenesis through oncogenic pathways. Additional regulators include farnesyltransferase, which anchors vesicular trafficking proteins to membranes; cholesterol synthesis, which sustains membrane curvature and rigidity; and syntenin, an adaptor protein that stabilizes protein– protein interactions during vesicle formation

Target or Pathway	Mechanism of Action	Agents or Interventions	Notes	Reference
ESCRT-dependent pathway	Inhibits formation of intraluminal vesicles via disruption of ESCRT machinery	Sulfisoxazole; siRNA targeting HRS, STAM1, TSG101	Downregulates ALIX, VPS4B, Rab GTPases; reduces exosomal MHC class II release.	[115]
Syndecan-syntetin-ALIX axis	Disrupts ESCRT accesory signaling	Heparan sulfate analogs	Inhibits tumor cell proliferation and invasiveness.	[116]
ESCRT-independent (ceramide-dependent)	Inhibits neutral sphingomyelinase 2, blocking ceramide-mediated vesicle formation	GW4869; spiroepoxide; DPTIP	Reduces exosome release; DPTIP is more potent than GW4869; enhances anti–PD-L1 efficacy.	[117]
ABC transporter activity	Blocks lipid recycling essential for exosome formation and release	Glibenclamide	Inhibits ABC transporters involved in exosomal lipid handling.	[118]
ABCA3 transporter	Inhibits exosomal export; increases intracellular drug retention	Indomethacin	Enhances doxorubicin accumulation in tumor cells.	[119]
V-ATPase proton pump	Disrupts pH gradient necessary for TDE biogenesis and release	Lansoprazole, omeprazole	Targets pH regulation in the TME.	[115]
EGFR signaling	Inhibits EGFR-mediated pathways involved in exosome generation	Erlotinib	Blocks EGFR-dependent vesicle production.	[120]
Farnesyltransferase activity	Disrupts prenylation of small GTPases critical for vesicle trafficking	Manumycin A; tipifarnib	Tipifarnib modulates both ESCRT-dependent and independent pathways; downregulates PD-L1.	[121]
Cholesterol synthesis	Reduces membrane availability for vesicle formation	D-pantethine	Decreases cholesterol-dependent exosome production.	[122]
Cytoskeletal remodeling	Inhibits microvesicle release by targeting actin–myosin and membrane shedding pathways	Calpeptin (calpain inhibitor), Y27632 (ROCK inhibitor)	Enhances chemosensitivity; suppresses microvesicle formation.	[123]
Syntenin	Blocks protein-protein interactions essential for exosome release	RNA interference; C58 peptide	Inhibits syndecan–syntenin interaction; reduces migration and clonogenicity.	[124]
PD-L1 trafficking	Increases exosomal PD-L1 export	6J1 (triazine)	Alters PD-L1 localization.	[125]
	Decreases membrane-bound PD-L1	LSD1 knockdown; miR-16-5p overexpression	Restores T cell cytotoxicity	[126]
TDE secretion in drug-resistant tumors	Inhibits exosome production in drug-resistant cancer phenotypes	Ketoconazole	Effective in sunitinib- resistant renal carcinoma.	[127]

These strategies enhance immunotherapy by disrupting tumor-derived exosomal communication networks and increasingly integrate nanotechnology with cellular immunotherapy to overcome exosome-mediated immune suppression and resistance.

### Turning the enemy’s messenger into a weapon: exosomes as therapeutic carriers

In parallel to efforts aimed at halting exosomal spread, a contrasting but highly innovative approach leverages exosomes themselves as delivery vehicles for cytostatic drugs, RNA molecules, or engineered protein cargo. TDEs and immune cell–derived exosomes are being repurposed for targeted drug transport, exploiting their intrinsic tropism, stability, and biocompatibility. There are several clinical studies using cytostatic drugs-loaded exosomes derived from tumor cells for targeted cancer therapy, as Table 4 shows:

**Table 4:** Studies of engineered TDEs as carriers of cytostatic molecules

**Feature**	GlioblastomaExo‑PTX	ProstatePTX‑EVs	A549 Lungcancer cellsCisplatin-EVs
**Source**	Exosomes derived from tumor cells (U-87) were loaded witd PTX	Exosomes derived from Tumor cells (LNCaP/PC‑3) were loaded witd PTX	Exosomes derived from tde human breast cancer cell line MDA‑MB‑231 were loaded witd cisplatin to create a tderapeutic nanoplatform called CaCE
**Loading methods**	Incubation, sonication	Incubation and endocytic pathway	Endocytosis
**Cellular uptake**	Cell-targeting adhesion molecules like tetraspanins and integrins on the surface of exosomes	Endocytosis	The TDEs entered cancer cells mainly through membrane fusion and endocytic pathways
**Efficacy**	The results showed exosomes in combination with PTX killed approx. 40% of tumor cells versus control group (approx. 8%)	Enhanced prostate cell killing	This exosome delivery system improved the survival rate in LLC tumor-bearing mice compared to co-administration of cisplatin and blocking antibodies
**Reference**	[128]	[129]	[130]

CAR-derived exosome-based nanoparticles represent a synergistic chemoimmunotherapeutic platform, exemplified by the CAR-EDC system-CAR-macrophage exosomes enriched with CXCL10 and conjugated to SN-38 which enables targeted drug delivery while simultaneously activating CD8⁺ T cells and promoting M1 macrophage polarization for enhanced antitumor efficacy [131]. A wide range of exosomes are in trials for solid tumors: paclitaxel (PTX) loaded exosomes, CAR exosomes, and RNA loaded exosomes are being tested across lung, breast, pancreatic, and other cancers [132]. In essence, this strategy targets the tumor from within using chemical agents while simultaneously mobilizing the immune system from the outside through immune activation.

One of the most formidable biological barriers is the BBB that limits the uptake of many therapies to treat glioblastoma and other brain tumors. The implementation of nanotechnology in CAR-T cell therapy can enhance the effectiveness of these cancer treatments while reducing adverse side effects [133, 134]. The enhanced permeability and retention (EPR) effect simultaneously promotes the passive accumulation of nanoparticles within tumor tissues, as they extravasate through the leaky tumor vasculature into the interstitial space [135]. Engineered TDEs loaded with rapamycin efficiently cross the BBB, enhance intratumoral drug accumulation, suppress VEGF-driven angiogenesis, and significantly inhibit glioblastoma growth [136]. Beyond their role as drug carriers, exosomes derived from CAR T cells offer a complementary mechanism of action: rather than transporting external agents, they infiltrate tumors and deliver intrinsic cytotoxic molecules, directly disrupting tumor growth [137]. For example, exosomes from anti-HER2 CAR-T cells containing cytotoxic proteins like granzyme B and perforin, enabling direct antitumor effects within the brain TME [11].

## Shadows triumph where light misleads: cellular armies versus Exosomal tactics

**Fig. 2:**
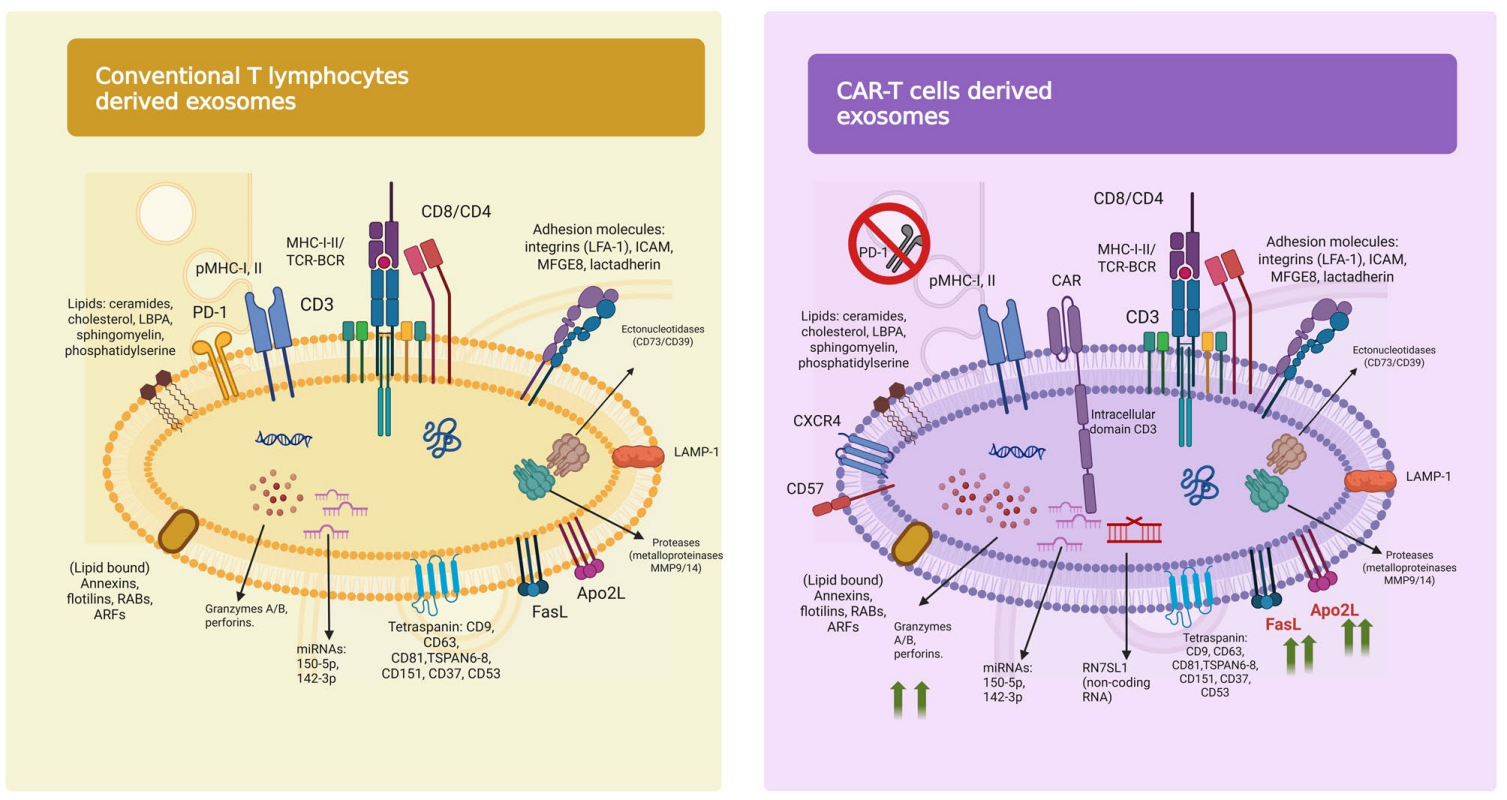
Structural and Molecular Composition of Conventional T Lymphocyte-Derived Exosomes vs. CAR-T Cell-Derived Exosomes

Exosome-based therapies are emerging as cell-free alternatives to adoptive cellular therapies (ACT), enabling the delivery of functional biomolecules while avoiding the severe toxicities of whole-cell approaches. Although ACTs such as CAR-T, TCR-T, and TIL therapies achieve high response rates—CD19 CAR-T reaching ~67% complete remission in relapsed acute lymphoblastic leukemia and 82% objective response in lymphoma [138, 139], and BCMA CAR-T achieving 73–84.6% overall response in multiple myeloma [140, 141], they are frequently limited by CRS, ICANS, GvHD, and poor penetration into the immunosuppressive TME [142, 143]. In this context, CAR-T cell–derived exosomes represent a promising cell-free strategy with potent antitumor activity mediated by receptor interaction, membrane fusion, and endocytic uptake [144–147]. These vesicles deliver a cytotoxic arsenal including perforin, granzymes (particularly granzyme B), FasL, Apo2L/TRAIL, and lysosomal enzymes, which trigger apoptosis through death receptor–mediated DISC formation, caspase-8 and caspase-3 activation, and Bid-dependent mitochondrial permeabilization [145, 148] **(**Fig. 2**)**. Although their effector mechanisms are largely antigen-independent, CAR expression confers tumor-specific targeting, enabling effective cytotoxicity even in tumors with low or heterogeneous antigen expression and potentially supporting transient antitumor immunity with reduced recurrence risk [115].

However, unlike memory CAR T cells, exosome-based therapies lack long-term persistence, and current evidence does not demonstrate that CAR T exosomes can prevent tumor reintroduction in the context of autologous CAR T cell therapy, thus requiring repeated infusions [149, 150]. CAR T exosomes are generated after antigen stimulation and can be isolated at high yield using GMP compatible procedures, with recovery rates of approximately 63% ± 5.7% for CD19 CAR–positive vesicles [151]. CAR T exosomes persist in circulation for up to two years after infusion, indicating sustained activity of their parental cells. Their protein cargo is enriched for granzyme B, perforin, and surface CAR molecules, which are absent in conventional T cell exosomes. HER2 CAR T exosomes contain at least 20-fold higher granzyme B levels than exosomes from unstimulated T cells (*p* < 0.001), and each CD19 CAR–positive exosome carries approximately 0.0005 µg of protein [152]. CAR T exosomes mediate antigen-specific cytotoxicity in vitro, producing dose-dependent killing of CD19-positive leukemia cells and inducing apoptosis in HER2-positive targets with caspase-3/7 activation comparable to CAR T cells but slower kinetics. In vivo, CAR T exosomes inhibit tumor growth with lower toxicity than CAR T cells and without cytokine release syndrome. Quantitatively, circulating CD19 CAR–positive vesicles eliminated 20.3% ± 3.8% of Raji cells and 37.8% ± 7.0% of SUP-B15 cells at 0.015 µg of vesicular protein per target cell, and mesothelin-targeted CAR exosomes reduced 4T1-MSLN cell viability to below 60% after 12 h [153]. Modeling estimates that circulating CD19 CAR–positive vesicles could eliminate 2.7 × 10¹¹ to 1.67 × 10¹³ tumor cells per day. By contrast, exosomes from non-CAR T cells showed negligible cytotoxicity, with killing rates of 0.71% ± 2.19% in CD19-positive cell lines and 2.35% ± 1.91% in primary CD19-positive cells [151]. These quantitative differences position CAR T exosomes as a potent and specific cell-free platform for targeted cancer therapy, with substantially greater cytotoxic activity and a more favorable safety profile than conventional T cell exosomes.

The figure highlights the most significant differences in CAR-T cell–derived exosomes, which are as follows: First, the absence of the PD-1 receptor, which allows these exosomes to evade neutralization by tumor cells upon binding to PD-L1. Second, they exhibit a higher abundance of granzymes and perforins, enhancing their tumor-destructive potential. Third and finally, CAR-T–derived exosomes uniquely express the CAR receptor, enabling precise tumor targeting with high specificity. It also shows key differences in lipid composition (e.g., ceramides, cholesterol, LBPA), membrane-associated proteins (e.g., annexins, flottins, RABs, ARFs), and cargo molecules (e.g., MHC-II/TCR-BCR complexes, adhesion molecules like LFA-1 and ICAM) between conventional T lymphocyte-derived exosomes and CAR-T cell-derived exosomes. Notable markers include CD3, CD4/CD8, CD63, CD81, CD57 and immune checkpoint molecules (e.g., PD-1). Abbreviations: LBPA (Lysobisphosphatidic Acid), MHC-II (Major Histocompatibility Complex Class II), TCR (T-Cell Receptor), BCR (B-Cell Receptor), LFA-1 (Lymphocyte Function-Associated Antigen-1), ICAM (Intercellular Adhesion Molecule), MFGE8 (Milk Fat Globule-EGF Factor 8), LAMP-1 (Lysosomal-Associated Membrane Protein-1), TSPAN-6 (Tetraspanin-6), PD-1 (Programmed Death-1), ARF (ADP-Ribosylation Factor), RAB (Ras-Associated Binding Protein). Created with BioRender.com.

Cellular immunotherapies exhibit immunogenicity and toxicity profiles that depend on cell origin and in vivo expansion: while allogeneic products can induce host–versus–graft reactions, currently approved autologous CAR T cells trigger severe toxicities such as CRS and ICANS as a consequence of antigen-driven activation and clonal expansion rather than host rejection. Despite their high efficacy in hematologic malignancies, CAR T cells show limited activity in solid tumors due to antigen heterogeneity, immunosuppressive TME, and off-tumor toxicity risk, prompting strategies such as improved antigen selection, accelerated manufacturing, frontline deployment, lymphodepletion, and locoregional delivery [154–156]. In contrast, exosomes display intrinsically low immunogenicity and toxicity owing to their endogenous nanoscale nature, immune-evasive lipid bilayer, protected cargo, and ability to penetrate biological barriers [157–161]. Unlike cellular therapies, exosomes do not proliferate or differentiate in vivo, eliminating risks of uncontrolled expansion and cytokine surges [162–164]. Preclinical models demonstrate that CAR T cell–derived exosomes retain potent antileukemic cytotoxicity without inducing systemic cytokine elevations or weight loss, and early clinical studies of exosome-based therapies report favorable safety profiles without immune hyperactivation, positioning exosomes as a safer, cell-free immunotherapeutic alternative [11, 151, 165]. Exosomes are non-replicating, acellular particles that lack the machinery to expand or secrete pro-inflammatory cytokines, which account for their favorable safety profile compared to ACT. Although some exosomes express MHC-peptide complexes and costimulatory molecules, their antigen presentation is often indirect, relying on uptake by host antigen-presenting cells (APCs). Without prolonged immune synapses or autonomous activation, exosomes trigger limited T cell expansion and minimal systemic inflammation [166].

Unlike CAR-T cells, CAR-T cell–derived exosomes lack PD-1, rendering their cytotoxic activity resistant to PD-L1–mediated suppression within the TME. These properties enable exosomes to achieve effective intratumoral delivery with reduced off-target toxicity, supporting their emerging role as versatile drug carriers, immunotherapeutic agents, and even cancer vaccines, in contrast to CAR-T cells that rely on physical migration and are frequently constrained by stromal barriers [167–176]. This functional versatility has enabled the extension of exosome-based strategies beyond direct cytotoxicity toward antigen delivery and cancer vaccination. In this context, exosomes derived from induced pluripotent stem cells (iPSC) function as carriers of tumor-associated antigens, overcoming the tumorigenicity and logistical limitations of direct iPSC administration [177]. A leading strategy involves loading iPSC-derived exosomes onto dendritic cells to generate vaccines, which in preclinical melanoma models induces efficient DC maturation, enhances T cell expansion 3.3–3.5-fold over controls, increases CD8⁺ T-cell infiltration, activates NK cells, reduces regulatory T cells in the tumor microenvironment, and translates into both prophylactic and therapeutic benefits, with survival rates reaching 70% in preventive and 60% in established tumor settings [178].

**Fig. 3:**
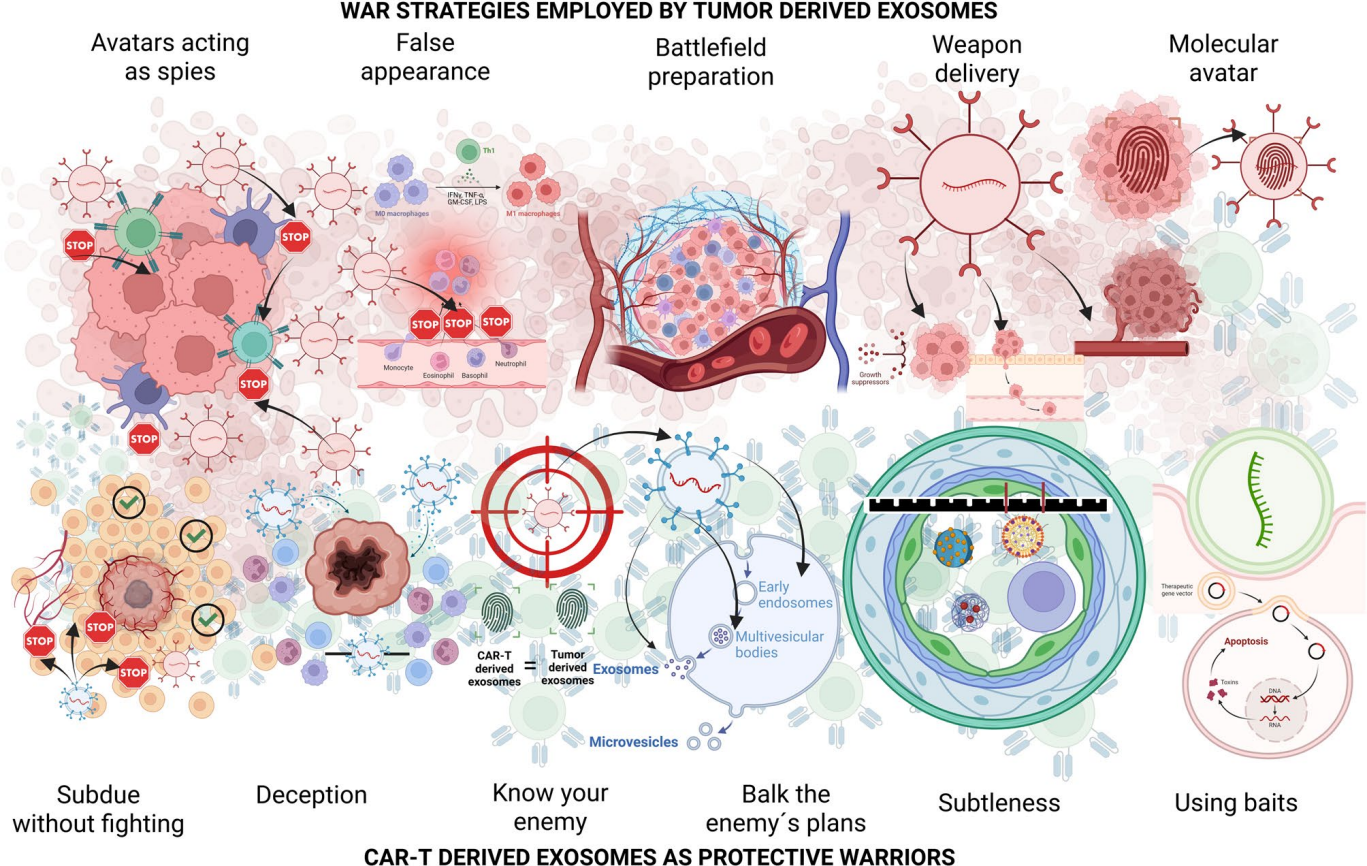
A complex interplay between Sun Tzu’s “Art of War” and oncologic treatment applications of exosomes

Exosomes offer a strategic advantage in ACT by enhancing antigen presentation. DC loaded with TDEs represents a complementary immunotherapeutic strategy by presenting a broad repertoire of tumor-associated antigens and inducing cytotoxic T lymphocyte responses. Recent models in glioma, leukemia, and hepatocellular carcinoma have shown that DC and TDE vaccination not only reshapes the immune landscape enhancing IFN-γ and reducing IL-10/TGF- β but also promotes long lasting antitumor T cell responses. This immune activation may synergize with CAR-T cell therapies by improving persistence and functionality. Moreover, combining CAR-T cell derived exosomes with DC vaccination platforms, particularly those leveraging engineered TDEs or iPSC-derived exosomes, could address antigen heterogeneity and tumor relapse [179, 180]. Summary of key comparisons are shown in Table 5. From a strategic standpoint, early intervention is essential: exosome-based approaches are most effective when implemented before tumor escape programs become fully established. Initiating these therapies during the early phases of microenvironmental remodeling increases the likelihood of durable responses and long-term disease control, reflecting the broader principle that complex challenges are best addressed before they fully manifest. See Fig. 3.

**Table 5:** Summary of key comparisons ACT versus exosomal therapy

Aspect	Cellular Therapy	Exosomal therapy	Exosome advantage
**Active agent**	Living cells	Cell-derived vesicles	No replicating cells; no risk of secondary T cell neoplasms, in contrast to the potential tumorigenic events reported with cellular CAR T products.
**Mechanism of action**	Direct cell killing via antigen recognition; in vivo cytokine release	Delivery of cytotoxic proteins/miRNAs; antigen presentation; drug/RNA delivery	Versatile cargo delivery; can combine mechanisms
**Tumor targeting**	Good antigen specificity; poor solid-tumor penetration	Can be engineered with targeting ligands; cross barriers	Better tissue penetration; engineerable tropism
**Safety/Side effects**	Risk of CRS, neurotoxicity, on-target/off-tumor effects	Generally safe; minimal systemic toxicity observed	Far lower toxicity; no CRS or GvHD
**Regulatory status**	Established	Nascent	Off-the-shelf potential once standardized
**Scalability/production**	Complex autologous culture	Can use cell lines/bioreactors; scalable purification (GMP processes emerging)	Potential for large-scale batch production
**Cost**	Extremely high per patient	Expected lower (mass-produced; no patient-specific labs)	Economies of scale; reuse of a standard product
**Storage/Handling**	Requires cryopreservation of live cells; limited shelf-life	Cryo- and lyophilization-stable; long shelf-life	Easier transport/storage; room-temp stability possible
**Clinical evidence**	Proven and approved in hematologic malignancies	Early-stage trials; some small studies of DC-exosomes show safety and disease stabilization; ongoing trials for drug delivery	Emerging but promising

War strategies employed by tumor-derived exosomes: (Upper side, from left to right) (1) Avatars acting as spies: Tumor-derived exosomes (TDEs) act as stealthy emissaries of malignancy, transferring oncogenic and immunosuppressive cargo that reprograms immune and stromal cells in distant niches, preparing the ground for immune evasion and metastasis. (2) False appearance: TDEs are camouflaged in a double lipid membrane, shielding their contents from immune detection and proteolytic degradation, while presenting a composition that mimics non-threatening or physiological signals. (3) Battlefield preparation: By reshaping the tumor microenvironment, inducing Treg and MDSC proliferation, and polarizing macrophages to the M2 phenotype, TDEs convert immune battlefields into sanctuaries of tolerance. (4) Weapon delivery: TDEs are equipped with immunosuppressive molecules, miRNAs, cytokines, and resistance-mediating proteins such as PD-L1 and ABC transporters, disabling effector cells and countering therapy. (5) Molecular avatar: Their proteomic and genomic content mirrors their tumor of origin, embodying a molecular extension of the malignant self, and creating decoys for immune responses

CAR-T derived exosomes as protective warriors: (Bottom side, from left to right): (1) Subdue without fighting: Synthetic or CAR-T-derived exosomes block tumor strategies such as angiogenesis and immune suppression while minimizing systemic toxicity, reshaping the immune landscape without provoking collateral damage. (2) Deception: By mimicking tumor-derived vesicles, therapeutic exosomes infiltrate malignant tissues undetected, delivering cytotoxic payloads such as perforin and granzyme B directly into the tumor core.

3. Know your enemy: Engineered CAR-T exosomes exploit molecular mimicry to engage tumoral receptors, enhancing selective targeting and cytotoxicity without PD-1-mediated inhibition.4. Balk the enemy’s plans: CAR-T exosomes are being designed to interfere with exosome biogenesis (e.g., via inhibition of the ESCRT pathway or ceramide synthesis), halting TDE production at its source. 5. Subtleness: Their nanoscale size (~80 nm) allows CAR T exosomes to evade immune surveillance and deeply penetrate solid tumors, unlike bulkier cellular therapies. 6. Using baits: By resembling TDEs or incorporating ligands for tumor receptors, therapeutic exosomes act as Trojan horses—delivering cytotoxic or gene-editing cargo (e.g., CRISPR/Cas9) to malignant cells or redirecting TDEs to safe elimination zones. Abbreviations: TDEs – Tumor-Derived Exosomes. CAR-T – Chimeric Antigen Receptor T cell. TME – Tumor Microenvironment. Treg – Regulatory T cell. MDSC – -Derived Suppressor Cell. PD-L1 – Programmed Death Ligand 1. PD-1 – Programmed Death 1. ABC transporters – ATP-Binding Cassette transporters. ESCRT – Endosomal Sorting Complex Required for Transport. CRISPR/Cas9 – Clustered Regularly Interspaced Short Palindromic Repeats/CRISPR-associated protein 9. miRNA – MicroRNA. Created with BioRender.com.

## Fire devours; smoke deceives: engineering exosomes and nanotechnology in cancer therapy

**Fig. 4:**
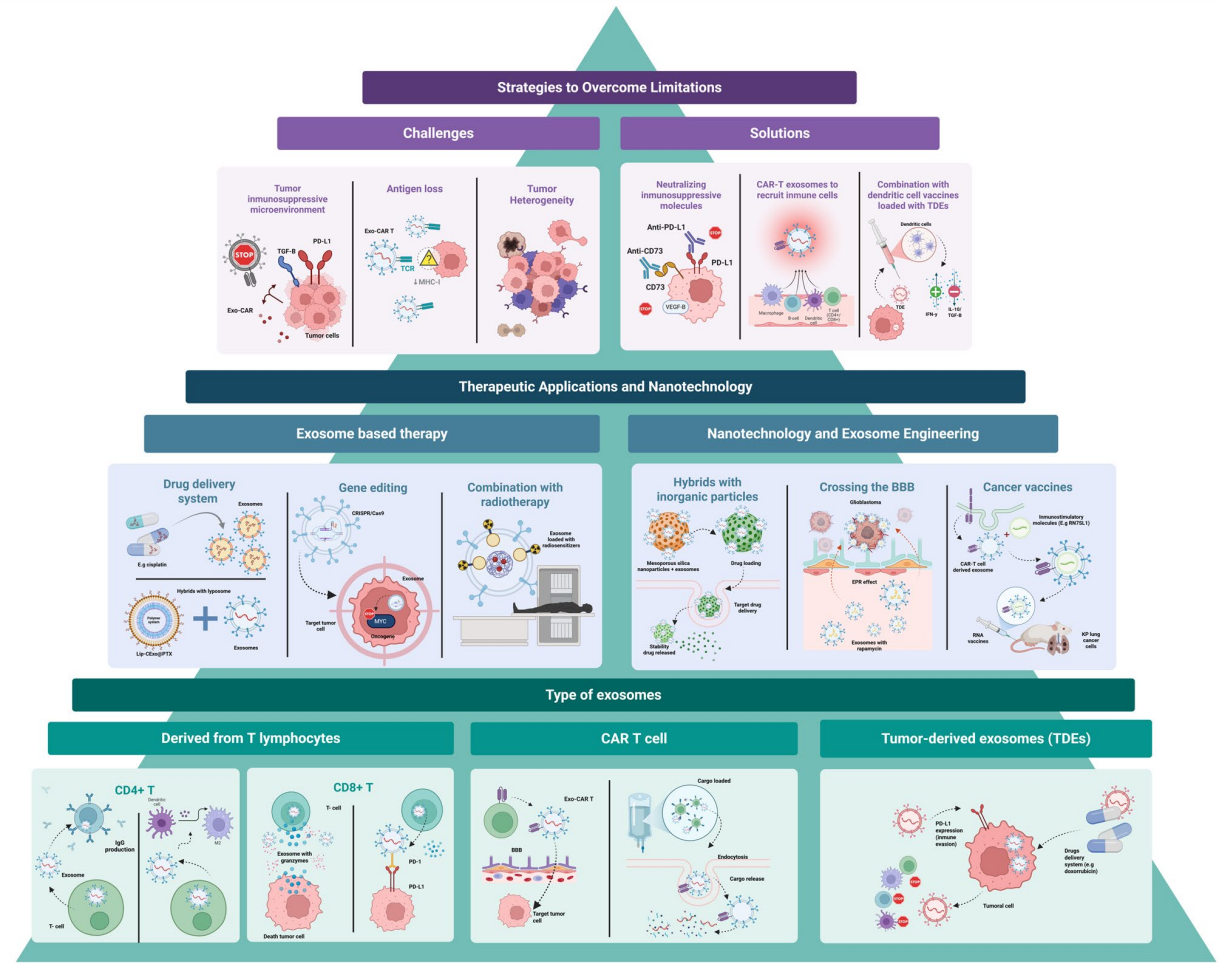
Exosomes, Nanotechnology, and Therapeutic Strategies in Cancer Treatment

Although CAR-T therapy is highly effective, its efficacy is limited by the TME, antigen heterogeneity and loss, and physical barriers such as hypoxia, acidosis, and extracellular matrix density. These constraints have driven the development of interdisciplinary strategies that integrate cellular therapies with exosomes and synthetic nanoparticles, which offer biocompatible, targeted platforms for delivering tumor antigens, co-stimulatory signals, and immunomodulatory cargos [133, 181, 182]. Together with polymeric and lipid-based nanocarriers, these engineered systems enhance CAR-based therapeutic efficacy while reducing systemic toxicity, establishing a convergent nanotechnology–immunotherapy framework for improved antitumor responses [183, 184]. **(**Fig. 4**)**. A systematic comparison of the diverse strategies employed for engineering these and other therapeutic platforms is provided in Table 6.

The figure illustrates a pyramid in the background that symbolizes the progressive layers of knowledge gained over time. At its foundation lies the well-documented mechanisms of action of various exosome types, including their unique characteristics and potential therapeutic targets, which are intrinsically linked to their parent cells or lymphocytes. Building upon this foundational understanding, the mid-section highlights the therapeutic techniques currently in use, which leverage these mechanistic insights. At the pinnacle, the pyramid points toward the future of exosome research, where scientific efforts must address persistent therapeutic barriers and challenges to unlock new clinical applications.

It also illustrates the role of exosomes (e.g., tumor-derived exosomes, TDEs; CAR-T cell-derived exosomes) and nanotechnology in overcoming challenges such as tumor heterogeneity, immune evasion (e.g., PD-L1/PD-1 axis), and drug delivery. Key strategies include exosome engineering (e.g., CRISPR/Cas9 gene editing), combination therapies (e.g., radiotherapy, dendritic cell vaccines), and hybrid systems (e.g., liposome-exosome hybrids, mesoporous silica nanoparticles). Abbreviations: CAR (Chimeric Antigen Receptor), PD-1 (Programmed Death-1), PD-L1 (Programmed Death-Ligand 1), TGF-β (Transforming Growth Factor-beta), MHC-I (Major Histocompatibility Complex Class I), VEGF-B (Vascular Endothelial Growth Factor-B), IFN-γ (Interferon-gamma), IL-10 (Interleukin-10), RN7SL1 (Immunostimulatory RNA), BBB (Blood-Brain Barrier), EPR (enhanced permeability and retention effect). Created with BioRender.com.

Although no phase III trials of exosomes implementation in the treatment of solid tumors have been publicly reported, exosome‑related technologies have already achieved meaningful clinical translation in diagnostics across multiple malignancies, including lung, pancreatic, colorectal, ovarian, glioma, and hepatocellular cancers, as Table 6 illustrates the different strategies. A Phase II clinical program (iEXPLORE, NCT03608631) has provided clinical evidence supporting engineered exosomes as delivery systems for KRASG12D-specific siRNA in metastatic pancreatic ductal adenocarcinoma, demonstrating excellent tolerability and safety in Phase Ia/Ib following systemic administration of iExoKrasG12D derived from allogeneic bone marrow mesenchymal stromal cells [9, 185]. Preclinical studies further show synergistic antitumor activity when iExoKrasG12D is combined with anti–CTLA-4 therapy, supporting its ongoing evaluation in combinatorial immunotherapy strategies.

Additionally, these technologies have already demonstrated real-world effectiveness in diagnostics. A notable example is the ExoDxTM Prostate (EPI) liquid biopsy for prostate cancer, which improves the detection of disease and reduces unnecessary biopsies compared with conventional approaches. This success highlights the translational potential of exosome-based platforms and their future applications, as ongoing research continues to establish their relevance as a promising therapeutic strategy [186].

**Table 6:** Systematic comparison of therapeutic engineering platforms

Strategy	Key Advantages	Main Disadvantages	Primary Applications	Technical Maturity	Example(s)	References
CAR-T derived exosomes	Reduced toxicity vs. cellular therapy; tumor targeting	Limited cargo capacity; scalability; loss of multifunctionality; batch variability	Overcoming antigen heterogeneity; reversing immunosuppression; gene delivery	Preclinical	RN7SL1-loaded CAR-T exosomes for CD19+/CD19 − tumors	[187]
Exosome-liposome hybrids	Enhanced drug loading; improved stability/circulation; dual targeting	Complex manufacturing; regulatory hurdles; higher cost; storage stability	Solid tumors with barriers; combination chemo-immunotherapy	Advanced Preclinical	LipCExo@PTX with anti-MSLN/PD-L1 scFv for lung cancer	[188]
Exosome-MSN hybrids	High payload; controlled/sustained release; imaging; tumor mimicry	Immunogenicity of inorganic components; rapid clearance; complex synthesis	Breast cancer; chemo-phototherapy; cytokine delivery	Preclinical	DOX-ICG loaded ID@EMSNs; IL-2/TGF-β co-loaded MSNs	[189]
Engineered TDEs for radiotherapy	Natural tumor homing; radiosensitizer delivery; amplify ROS	May promote resistance; cargo changes post-radiation; bystander effects	Radiosensitization; overcoming radioresistance	Early Preclinical	Manganese/FeS2 nanomaterial-loaded TDEs	[190]
MHC-engineered TDEs	Activation of CD8+/CD4 + T cells; broad immune response; antigen presentation	Requires genetic modification; MHC-II engineering complexity; autoimmunity risk	Therapeutic vaccination; personalized immunotherapy	Preclinical	MHC-I/II dual-presenting TDEs	[191, 192]

This table summarizes the major exosome-based strategies, highlighting their key advantages, disadvantages, primary applications, technical maturity, and representative examples. Most approaches remain in preclinical or early translational stages, with scalability, regulatory, and safety challenges as common barriers to clinical adoption.

### Where thought does not reach, let your form emerge: CAR-T exosomes as specialized warriors

Although CAR-T–derived exosomes retain intrinsic tumor-targeting specificity, they can be further engineered to enhance safety and efficacy [183], including the delivery of CRISPR/Cas9 systems against oncogenic drivers such as MYC. In Raji xenograft models, CAR-T exosomes loaded with MYC-targeting sgRNA/Cas9 plasmids effectively suppressed tumor growth, highlighting their therapeutic potential. Compared with viral vectors, exosomes offer lower immunogenicity, reduced carcinogenic risk, and improved cargo encapsulation efficiency [193]. Given that MYC is dysregulated in approximately 30% of human cancers and is strongly associated with poor prognosis, exosome-mediated gene editing represents a promising strategy for targeting this critical oncogenic pathway [12].

mRNA-engineered exosomes offer a virus-free alternative to traditional lentiviral CAR-T manufacturing, allowing fast, temporary, and well-controlled CAR expression. The CAR-encoding mRNA can be loaded into exosomes from inside the producing cells using plasmid transfection or RNA-binding proteins (such as MS2P, HuR, and L7Ae), or from outside using techniques like electroporation, nanoporational loading, cationic lipids, or liposome fusion, which can increase loading efficiency up to 1000-fold. These methods enable efficient transfer of CAR mRNA into T cells, reducing dependence on viral vectors and even allowing CAR-T generation directly in vivo [194, 195]. In addition, CAR-T–derived exosomes can be combined with RNA vaccines such as RN7SL1 (Ova-19-7SL), an endogenous RNA that activates innate immune sensors (RIG-I and MDA5) to boost CAR-T expansion and function. In mouse models with mixed CD19⁺ and CD19⁻ lung tumors, RN7SL1-loaded CAR-T exosomes enhanced CAR-T activity, activated other immune cells in the TME, reversed immunosuppression, and significantly reduced tumor growth [187]. Together, these results show that multifunctional CAR-T exosome engineering can help overcome antigen heterogeneity, strengthen both innate and adaptive immunity, and improve CAR-T therapy in solid tumors while potentially counteracting tumor-driven immune resistance [196].

### The humble Reed sways to the unseen wind: Exosome–liposome and exosome–nanoparticle hybrids as engineered weapons in cancer therapy

Inorganic nanoparticles can be engineered for high drug payload, controlled release, and imaging capabilities, but may have issues with immunogenicity and rapid clearance. Hybridizing exosomes with inorganic nanoparticles can improve drug encapsulation efficiency, stability, and targeted delivery, while reducing off-target effects and systemic toxicity [189].

Hybrid nanovesicles generated by fusing CAR-T–derived exosomes with lung-targeted liposomes represent a highly effective multifunctional therapeutic platform. In murine lung cancer models, bispecific CAR-T exosomes loaded with paclitaxel (Lip-CExo@PTX) achieved > 95% lung accumulation and induced rapid tumor regression. Incorporation of an anti-mesothelin (MSLN) scFv enabled selective delivery of PTX together with cytotoxic effectors such as granzyme B and perforin to MSLN-positive tumors, while a PD-L1–blocking scFv prevented immune checkpoint–mediated inhibition, thereby enhancing immunogenic cell death and antitumor immunity. In metastatic CT-26 lung cancer, Lip-CExo@PTX significantly prolonged survival, demonstrating that engineered exosome–liposome hybrids can function as precisely coordinated, high-efficacy chemoimmunotherapeutic systems [188, 193].

Also, mesoporous silica nanoparticles (MSNs) have emerged as versatile drug-delivery platforms. Tian et al. developed an exosome-coated MSN system (ID@E-MSNs) using 4T1-derived exosomes to mimic tumor cells, enhancing tumor accumulation and enabling synergistic chemo-photothermal therapy through co-delivery of doxorubicin and indocyanine green. In parallel, Liu et al. designed MSNs for controlled co-release of IL-2 and TGF-β, which strengthened antitumor immunity by promoting terminal effector T-cell differentiation and reshaping the TME through vascular normalization and increased T-cell infiltration [197].

Different methods regarding drug delivery with EV have been explored. Engineering approaches generally fall into two categories: surface modification to enhance targeting specificity and cargo modification to increase biological activity. By combining the intrinsic targeting capacity, immunomodulatory properties, and biocompatibility of exosomes with the tunable functionalities of liposomes or other nanoparticles, hybrid systems can achieve therapeutic effects that surpass those of natural vesicles alone. These exosome nanoparticle hybrids not only enhance cargo delivery and cellular uptake but also enable multifunctional platforms combining imaging, controlled release, and immunomodulation in a single vehicle. To generate such hybrid systems, several surface-engineering strategies have been developed, including lipid–PEG–lipid insertion, glycan modification, covalent bioconjugation, and click chemistry-based conjugation [198].

Lipid–PEG–lipid insertion has enabled the formation of hybrid nanocarriers that fuse long-circulating liposomes with exosomes, creating platforms with improved pharmacokinetics for cancer therapy [199]. Incorporation of PEGylated lipids into exosomal membranes extends circulation time while preserving exosome biochemistry and biophysical integrity, and PEG-mediated fusion has become a widely used strategy to enhance exosome drug-delivery capacity [200, 201]. Because PEG-driven fusion remains limited, newer approaches employ Tat-PEG-lipids whose membrane-associating peptide (YGRKKRRQRRR) and lipid tail particularly myristoyl (C14) and dodecanoyl (C12) promote more efficient exosome–liposome fusion [202]. Although concerns persist regarding potential toxicity or immunogenicity of PEG and related polymers, these issues are increasingly viewed as engineering challenges, prompting systematic investigation into how membrane modifications influence exosomal “self” identity and surface protein composition [203]. Collectively, growing in vitro and in vivo evidence supports the refinement of hybrid exosomes as complex, tunable nanocarriers with expanding therapeutic promise.

Surface insertion and lipid anchoring provide modular strategies to functionalize exosomal membranes while preserving their native architecture. In lipid-PEG–ligand post-insertion, functionalized PEG-lipids carrying maleimide, biotin, or azide groups are introduced into liposomes and then transferred to exosomes, enabling the addition of targeting ligands, stealth coatings, or stimuli-responsive elements with minimal structural disruption. Hydrophobic tail-anchored ligands such as cholesterol- or long-alkyl–conjugated peptides integrate spontaneously into the exosomal bilayer, offering rapid functionalization although they may influence membrane packing and protein mobility [198].

Covalent bioconjugation and click chemistry enable precise, stable, and site-specific exosome surface modification, typically by attaching targeting ligands to surface protein amines through copper-catalyzed or strain-promoted azide–alkyne cycloadditions [204]; using this strategy, Jia et al. enhanced glioma targeting via CuAAC [205]. More traditional chemistries, including maleimide–thiol and EDC/NHS coupling, offer scalable routes for modifying abundant amine and thiol groups, though they risk heterogeneous conjugation. Enzymatic ligation methods such as sortase a provide true site specificity while preserving membrane integrity, but at the cost of greater technical complexity [198].

Combining methods such as extrusion-based fusion, PEG-lipid insertion, and final covalent ligand attachment can significantly enhance hybrid exosome performance, as long as the steps are applied in the correct order to preserve membrane integrity and previously added components. These engineered exosome–nanoparticle hybrids surpass natural exosomes by offering adjustable pharmacokinetics, multimodal cargo delivery, and controlled therapeutic activation, making them a flexible platform for personalized therapies that integrate targeted cytotoxicity, immune modulation, and real-time imaging.

### Like water, shaping nothing, becoming formless: tumor exosomes in radiotherapy

Recent studies highlight the dual role of TDE in radiotherapy: Not only do they mediate radioresistance and contribute to radiation induced bystander effects, but they also present a promising therapeutic target for improving treatment efficacy. Yang et al. demonstrated that irradiation significantly increases exosome biogenesis and release, and modulates their cargo such as DNA fragments and miRNAs, that can shuttle DNA damage signals and survival promoting messages to both irradiated and adjacent non-irradiated cells. These insights suggest that disrupting exosomes-mediated communication could enhance radiosensitivity or selectively target tumor cells. Building on this, engineered exosomes can be loaded with radiosensitizers or immune adjuvants to counteract TDE-mediated survival pathways, directly linking radiotherapy strategies to exosome-based therapeutic engineering. The TDEs can be engineered to carry radiosensitizer, such as manganese-based nanomaterials, which amplify reactive oxygen species (ROS) production upon irradiation, thereby increasing DNA damage in tumor cells. Other strategies include loading exosomes with sodium iodide symporters to improve uptake or combining exosomes with immune adjuvants to reverse local immunosuppression [115, 190].

At the same time, more recent findings have revealed specific molecular mechanisms by which exosomes contribute to radioresistance and how these can be therapeutically reversed. For instance, in breast cancer, irradiation has been shown to co-induce CD47 and HER2 expression, promoting immune evasion and tumor proliferation [206]. The blockade of both targets results in reduced clonogenic survival and enhanced macrophage-mediated clearance of tumor cells. In parallel, nanotechnology-based interventions have utilized exosome membranes to deliver FeS2-based oxidative stress amplifiers, which selectively target tumors and enhance low-dose radiotherapy by increasing hydroxyl radical generation. These advanced strategies further underscore the dual potential of TDE as mediators of resistance and as tools to sensitize tumors to radiotherapy through targeted delivery systems [207].

Radiation therapy can induce profound alterations in the TME, largely through epigenetic remodeling. Off-target radiation effects may promote aberrant DNA methylation, while exosomes released from irradiated cells often contain modified molecular cargo that can be horizontally transferred to non-irradiated cells. Several exosomal microRNAs have been shown to dampen the activity of immune effector populations such as NK cells thereby facilitating tumor progression, adaptive responses to radiation, and late recurrences long after the initial therapeutic exposure. These vesicles act as molecular proxies, extending the influence of irradiated cells beyond their physical boundaries, subtly shaping the biological terrain [208].

Radiation exposure also triggers extensive histone modifications, including ubiquitination, phosphorylation, acetylation, and methylation, ultimately altering chromatin structure and gene expression. Such epigenetic disruptions contribute to ionizing-radiation resistance, and exosomes can disseminate these regulatory cues to neighboring, non-irradiated cells, allowing the adaptive phenotype to propagate through the TME much like a silent strategic maneuver [209].

Non-coding RNAs within exosomes released from irradiated cells can further promote EMT through activation of β-catenin, Notch, and Smad2/3 signaling pathways, enhancing the malignant behavior of recipient cells. These exosomal long non-coding RNAs also hold potential as biomarkers for prognostic surveillance and post-irradiation therapeutic monitoring [210, 211].

Hypoxic tumor cells—intrinsically more radioresistant than oxygenated cells—exhibit an increased release of exosomes and significant alterations in their cargo. Hypoxia-derived exosomes can stimulate angiogenesis through shuttling of molecules such as miR-135b, which modulates components of the HIF-1 axis [212]. Furthermore, these vesicles can reduce radiation-induced apoptosis and enhance DNA-damage repair, conferring radioresistance via transfer of miR-340-5p to recipient normoxic cells. In this way, hypoxic cells deploy exosomal signals that reinforce survival pathways and recalibrate the microenvironment, enabling the tumor to endure therapeutic pressures with a quiet but deliberate precision [213].

TDEs promote radioresistance through defined mechanisms, including increased DNA repair signaling, transfer of survival-associated microRNAs, metabolic reprogramming, and immune evasion driven by CD47 or HER2. These pathways constitute actionable engineering targets for exosome and nanotechnology platforms. Exosomes derived from CAR T cells can be engineered to deliver CRISPR Cas9 cargos that disrupt genes associated with radiation tolerance, such as MYC or CD47, or to transport immunostimulatory RNAs, including RN7SL1, to counteract the immunosuppressive environment intensified by radiotherapy. Hybrid systems that combine exosomes with liposomes or inorganic nanoparticles can be designed to co-deliver radiosensitizers, ROS amplifiers, or scFv that block immune checkpoints within irradiated tumors, this approach involves the dual action of inhibiting TDE-mediated pro-survival signaling while exploiting the enhanced tumor permeability resulting from radiation exposure. In this framework, engineered exosomes and synthetic hybrids function as targeted countermeasures against the molecular circuits underlying TDE-driven radioresistance.

## The purest honey yields but drops: limitations of exosomes as therapeutic agents

Several cellular therapies are FDA-approved, such as CAR-T for leukemia/lymphoma or TIL for melanoma, with defined regulatory pathways [214, 215]. In contrast, exosome therapies are largely experimental. Currently there is an increasing number of clinical trials investigating exosome-based therapies in solid tumors, but most are still in Phase I/II [216]. Exosome-based products do not yet have established regulatory guidelines, and agencies are still discussing how to classify and standardize them [161, 217]. Challenges include heterogeneity of vesicle populations and lack of standardized isolation. Recent work is addressing these technical gaps by introducing tangential flow filtration and developing stable, well-characterized producer cell lines for clinical-grade exosome production [218, 219].

Although some manufacturing steps may become more cost-efficient, regulatory barriers remain substantial. Because exosome therapies are acellular and non-replicative, they fall outside the ATMP classification yet are often regulated under frameworks designed for live-cell products in the United States and European Union. This mismatch imposes disproportionate requirements for characterization, GMP manufacturing, and preclinical testing. In the United States, for example, the FDA regulates exosome products as biologics under the Public Health Service Act and has issued safety warnings against unapproved exosome-based interventions marketed directly to patients [220].

In Europe, the EMA’s Committee for Advanced Therapies (CAT) assesses exosome products individually, and they are not classified as ATMPs unless they contain genetic modifications [221]. Nonetheless, manufacturers are often required to meet ATMP-level GMP, biodistribution, immunogenicity, and sterility standards, increasing cost and slowing clinical development. This regulatory ambiguity has also encouraged a gray market of unregulated exosome products marketed as cosmetics or wellness supplements, prompting safety warnings from agencies such as the FDA.

Accordingly, regulators and scientific societies are advocating for exosome-specific guidelines that reflect their acellular nature and establish standardized manufacturing and quality criteria. Initiatives toward such frameworks are underway in the United States, Japan, and international consortia, but exosome therapies still remain in an early and evolving regulatory stage compared with the more established pathways governing cellular therapies (**see** Table 8) [7, 222, 223].

Personalized cell therapies are costly and operationally complex: autologous CAR-T products require individualized cell collection, 2–3 weeks of expansion, strict sterility control, and extensive coordination, resulting in 17–22 days manufacturing times and costs of $300,000–$500,000 USD per patient [224, 225]. Allogeneic platforms aim to reduce this burden but still face rejection risks and demanding bioprocessing requirements.

In contrast, exosomes can be generated from standardized producer cell lines, enabling an off-the-shelf model [219]. Scalable GMP production using bioreactors, tangential flow filtration, and chromatography has already been demonstrated, and exosomes remain stable for months even after lyophilization greatly simplifying storage, distribution, and overall logistics compared with living cell therapies [66].

Despite their remarkable potential in drug delivery and immunomodulation, exosomes face significant challenges that hinder their clinical translation [226]. One of the foremost limitations lies in the standardization of isolation and purification techniques. Conventional methods such as ultracentrifugation, size exclusion chromatography, and precipitation kits often result in heterogeneous populations with varying purity and yield, leading to inconsistencies in downstream applications [7]. Moreover, cross-contamination with other EVs or soluble proteins can alter their therapeutic efficacy and safety profile [227, 228]. Without robust, reproducible, and scalable isolation methods, the reliability of exosome-based products remains questionable.

Another critical barrier is the lack of control over exosomal cargo loading and retention. Both passive and active loading methods like electroporation, sonication, or chemical transfection exhibit low efficiency, potential structural damage to the vesicles, and often result in suboptimal therapeutic payloads [228]. Furthermore, the cargo naturally enclosed during biogenesis is highly variable and depends on the physiological state and source of the parent cell, raising concerns about batch-to-batch variability [227, 229]. This unpredictability not only compromises therapeutic consistency but also introduces safety concerns, especially if the exosomes inadvertently carry oncogenic or pro-inflammatory molecules [229].

Biodistribution and targeting specificity remain unresolved issues in exosome therapy [230]. Although engineering approaches like ligand modification or surface protein fusion have been explored to enhance targeting, these methods are still in early development and lack validation in clinical settings [227]. Additionally, pharmacokinetics and biodistribution data are scarce, which complicates the design of optimal dosing regimens and safety evaluations [228].

To overcome these limitations and enable clinical scalability, future research must prioritize a deeper understanding of exosome biogenesis and cargo-sorting mechanisms, (**see** Table 7). Elucidating the molecular machinery that governs the selective packaging, retention, and release of bioactive molecules into EVs would significantly reduce cargo variability and improve therapeutic consistency [227, 228]. Additionally, advances in bioengineering strategies, including genetic or chemical surface modifications, could enhance tissue-specific targeting and mitigate off-target accumulation [227]. Efforts should also focus on developing GMP-compliant, scalable manufacturing platforms and robust in vivo tracking tools to ensure reproducibility and safety [228, 229]. Advances in bioreactor-based systems, artificial exosome synthesis, and emerging isolation technologies such as microfluidics and tangential flow filtration (TFF) are addressing issues of scalability and reproducibility [231]. Artificial intelligence (AI), including machine learning and deep learning, has demonstrated value in exosome biomarker discovery, diagnostic precision, and therapeutic optimization [232, 233]. Algorithms trained on diverse datasets can reduce bias and improve clinical generalizability, while AI model exosome TME interactions and in silico simulations of hybrid exosome design enabling precise ligand selection and optimized therapeutic cargo loading [234, 235].

**Table 7:** Critical bottlenecks in exosome therapeutics and emerging solutions

Bottleneck	Description	Emerging Solutions
Cargo Sorting and Loading	Inefficient, poorly understood mechanisms for selective cargo incorporation	Genetic engineering, advanced loading technologies [236]
In Vivo Tropism and Biodistribution	Unpredictable targeting, off target effects	Surface functionalization, ligand engineering [237]
Isolation and Purification	Low yield, heterogeneity, lack of standardization	Microfluidics, improved protocols, GMP compliance [238]
Regulatory Ambiguity	Fragmented global standards, unclear safety/efficacy requirements	Harmonization efforts, collaborative frameworks [239]
Preclinical vs. Clinical Potency	Discrepancy between animals and human outcomes	Enhanced targeting, robust clinical trial design [240]
Large-Scale Production and Stability	Scalability, storage, and reproducibility issues	Bioprocess optimization, AI-driven manufacturing [241]
Safety and Immunogenicity	Risk of immune response, infectious transmission	Advanced characterization, rigorous safety testing [242]

**Fig. 5:**
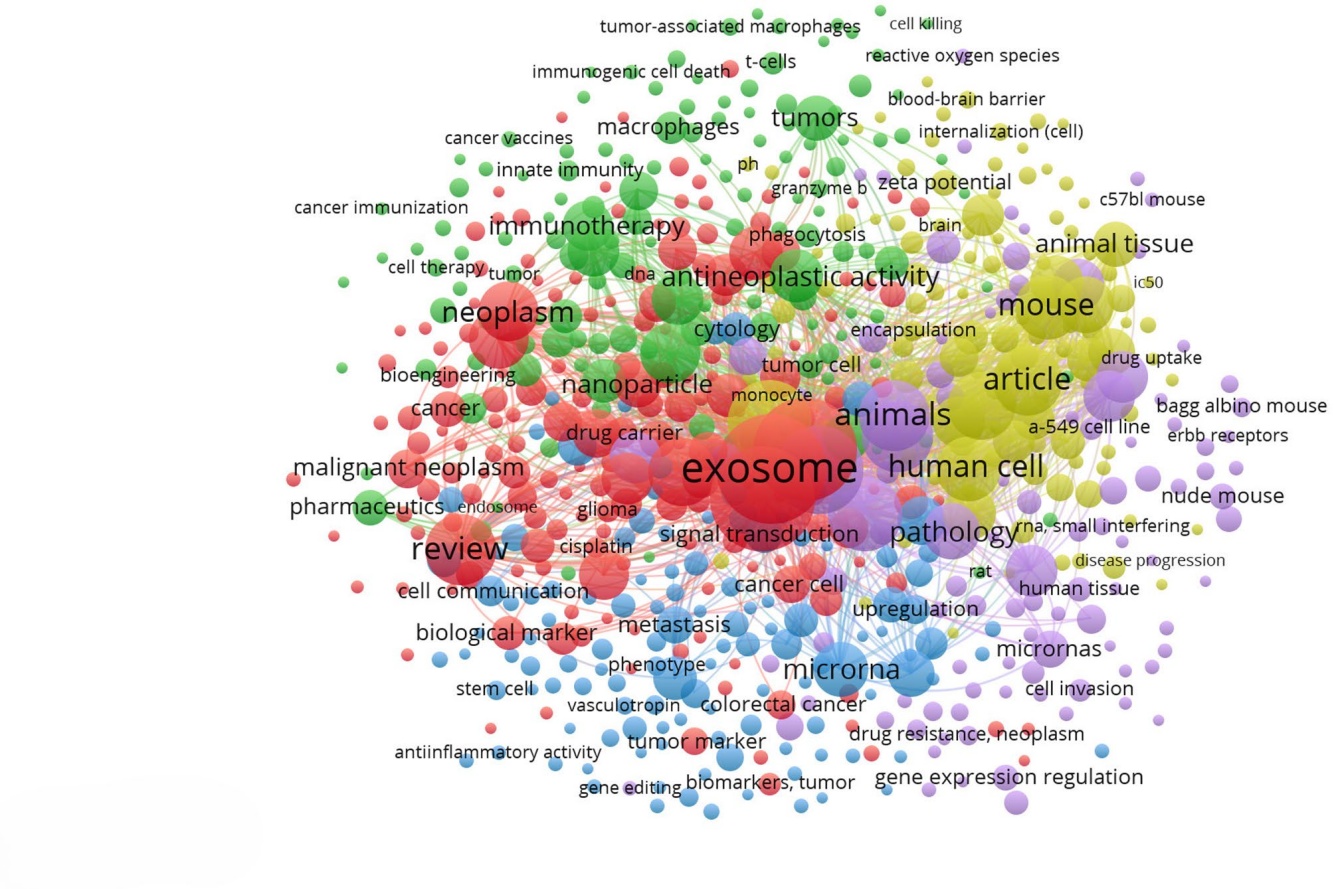
Co-occurrence network of author keywords related to exosome research in oncology

Although the U.S. FDA has not yet issued EV-specific regulatory guidance, recent scientific and technological advances offer practical paths to reduce current gaps [243]. A deeper mechanistic understanding and increasingly standardized engineering methods help define Critical Quality Attributes (CQAs) by extrapolating from existing biologic and cell therapy frameworks, while harmonized characterization standards such as ISEV-MISEV2023/2024 improve comparability across studies [67, 244]. In parallel, scalable manufacturing platforms including tangential flow filtration, microfluidics, and improved potency and release assays are becoming aligned with expectations outlined by EMA/CAT and CBER for complex cell-derived biologics, even in the absence of dedicated EV pathways. Together, these developments illustrate how the integration of nanotechnology, bioengineering, and AI is accelerating the clinical translation of exosome-based therapies and advancing their potential in next-generation cancer treatment (See Table 8). Our conceptual findings align with the principal research domains identified through bibliometric analysis of recent publications **(**Fig. 5**)**, which highlight exosomes as central nodes connecting tumor biology, immunotherapy, and nanotherapeutics. This convergence underscores the relevance of the strategies discussed, as they parallel the most active directions currently pursued by the scientific community.

**Table 8:** Comparative overview of regulatory landscapes for EV-based therapeutics

Region/Agency	Regulatory Classification	Existing Documents	Key Gaps/Challenges
United States (FDA/CBER)	No EV-specific classification. EV-based products are currently assessed by analogy to biologics, cell- and gene-based therapies.	-General CBER regulatory science initiatives for complex and cell-derived products -No EV-specific guidance.	-Absence of EV-specific quality, potency, identity, and manufacturing guidance. -Developers must extrapolate from cell/gene therapy rules, which do not fully address EV heterogeneity. -Lack of validated potency assays.
European Union (EMA/CAT)	Case-by-case assessment. Some EV-based products may fall under “biological medicinal products” per CAT criteria.	-EMA/CAT Reflection Paper on EV-based Medicinal Products. -General ATMP guidance.	-Reflection paper provides considerations but not prescriptive requirements. -Need for consensus on minimal characterization, CQAs, and release criteria.
Japan (PMDA)	Actively evaluating EV-based therapies; some products classified under regenerative medicine products.	-PMDA considerations for cell-derived and EV-derived products.	-Early-stage framework; limited detail on manufacturing controls or potency assays.
Global scientific standardization (ISEV – MISEV2023/24)	Not a regulatory classification.	- MISEV guidelines for reporting and characterization	- Reporting-only framework; does not define manufacturing or regulatory pathways.

The network was generated from *n* = 456 indexed publications (2020–2025) using VOSviewer (version 1.6.20). Each node represents an author keyword, with node size proportional to its occurrence frequency and edges reflecting co-occurrence strength (association strength normalization). Colors denote clusters identified by modularity-based clustering, revealing four major thematic domains: (i) exosome biology and tumor microenvironment (red), (ii) drug delivery and nanotherapeutics (green), (iii) immunotherapy and immune modulation (blue), and (iv) experimental models and translational studies (yellow/purple). The figure highlights the centrality of “exosome(s)” and “tumor microenvironment” as research hubs, with growing convergence toward therapeutic applications and translational models.

## Conclusion

Exosomes have emerged as both adversaries and allies in cancer biology and immunotherapy. TDEs, acting as molecular proxies of malignancy, orchestrate immune suppression, metastasis, and therapeutic resistance. Yet the same architecture that enables them to broadcast disruptive signals can be redirected toward therapeutic purpose. CAR-T–derived exosomes embody this turning point. As cell-free vectors, they bypass several constraints of conventional CAR-T therapy: their nanoscale dimensions allow efficient navigation of the dense tumor stroma, the absence of checkpoint receptors limits susceptibility to immunosuppressive cues, and their acellular nature minimizes risks of uncontrolled expansion or cytokine storm, enabling safer and more predictable dosing.

Advances in bioengineering and nanotechnology further refine these vesicles into deliberate therapeutic instruments. Engineered exosomes capable of delivering CRISPR/Cas9 systems, chemotherapeutics, or immunomodulatory RNAs illustrate the breadth of this platform. Hybrid constructs that integrate exosomes with liposomes or inorganic nanoparticles enhance tumor selectivity, reshape the TME, and amplify anti-tumor responses. In this sense, engineered exosomes operate as disciplined extensions of their cellular origin avatars crafted to act with the stealth, precision, and adaptability required to navigate cancer’s shifting terrain.

Challenges, however, remain substantial. Isolation and characterization methods lack standardization, often producing heterogeneous preparations that limit reproducibility and scalability. Cargo loading remains inconsistent, and control over biodistribution and pharmacokinetics is still incomplete. Regulatory frameworks provide no dedicated pathway for exosome-based products, forcing developers to adapt guidelines built for fundamentally different therapeutics. Addressing these gaps will require tightly integrated progress across molecular biology, bioprocess engineering, analytics, and regulatory science.

Despite these obstacles, the direction of the field is unmistakable. Exosomes are emerging as modular, programmable, and scalable therapeutic agents capable of adapting to the complexities of cancer. If TDEs represent the quiet advance of malignancy, CAR-T exosomes and engineered exosomes stand as their deliberate counterparts—subtle, strategic, and designed to restore equilibrium within the host. As mechanistic insight deepens and technological capability expands, these cell-free vectors may transition from passive biomarkers to decisive actors in cancer therapy. Echoing Sun Tzu’s principle that victory begins long before the battlefield is visible, the groundwork for outmaneuvering cancer is already being laid.

## Data Availability

Not applicable.

## Abbreviations

ABC transporters: ATP-Binding Cassette transporters
ACT: Adoptive Cell Therapy
ADC: Antibody–Drug Conjugate
ADAM: A Disintegrin and Metalloprotease
ALIX: Apoptosis-Linked Gene 2-Interacting Protein X
APC: Antigen-Presenting Cell
ARF: ADP-Ribosylation Factor
ATMP: Advanced Therapy Medicinal Product
ATPase: Adenosine Triphosphatase
BBB: Blood–Brain Barrier
BCR: B Cell Receptor
BCMA: B-Cell Maturation Antigen
BiTE: Bispecific T-cell Engager
BMSC: Bone Marrow–Derived Mesenchymal Stem Cell
CAR: Chimeric Antigen Receptor
CAR-T: Chimeric Antigen Receptor T cell
CAR-M: Chimeric Antigen Receptor Macrophage
CAT (EMA): Committee for Advanced Therapies
circRNA: Circular RNA
CNS: Central Nervous System
CQAs: Critical Quality Attributes
CRISPR/Cas9: Clustered Regularly Interspaced Short Palindromic Repeats / CRISPR-associated protein 9
CRS: Cytokine Release Syndrome
CuAAC: Copper-Catalyzed Azide–Alkyne Cycloaddition
CXCL10: C-X-C Motif Chemokine Ligand 10
DAF: Decay Accelerating Factor
DC: Dendritic Cell
DISC: Death-Inducing Signaling Complex
DLS: Dynamic Light Scattering
DNA: Deoxyribonucleic Acid
DOX: Doxorubicin
DR4/DR5: Death Receptor 4 / Death Receptor 5
DPTIP: Diperodon Tip Inhibitor of Neutral Sphingomyelinase 2 dsDNA/ssDNA – Double-Stranded DNA / Single-Stranded DNA ECM – Extracellular Matrix
EDC/NHS: 1-Ethyl-3-(3-dimethylaminopropyl)carbodiimide / NHydroxysuccinimide
EGFR: Epidermal Growth Factor Receptor
ELISA: Enzyme-Linked Immunosorbent Assay
EMA: European Medicines Agency
ESCRT: Endosomal Sorting Complex Required for Transport
EV/EVs: Extracellular Vesicle(s)
FasL: Fas Ligand
FDA: U.S. Food and Drug Administration
GPI: Glycosylphosphatidylinositol
GPCR: G Protein-Coupled Receptor
GvHD: Graft-versus-Host Disease
GZMB: Granzyme B
HLA/MHC: Human Leukocyte Antigen / Major Histocompatibility Complex
HRS: Hepatocyte Growth Factor–Regulated Tyrosine Kinase Substrate
HSPs: Heat Shock Proteins (Hsp60, Hsp70, Hsp90)
HuR/MS2P/L7Ae: RNA-binding proteins used for mRNA loading
ICAM-1: Intercellular Adhesion Molecule 1
ICD: Immunogenic Cell Death
ICG: Indocyanine Green
ICANS: Immune Effector Cell–Associated Neurotoxicity Syndrome
IFN-γ: Interferon Gamma
IL-2: Interleukin 2
ILVs: Intraluminal Vesicles
iPSC: Induced Pluripotent Stem Cell
iExoKrasG12D: Exosomes Loaded with siRNA Against KRAS G12D
LBPA: Lysobisphosphatidic Acid
LFA-1: Lymphocyte Function–Associated Antigen 1
lncRNA: Long Noncoding RNA
Lip-CExo@PTX: Liposome–CAR-T Exosome Hybrid Nanoparticles with Paclitaxel
LOX: Lysyl Oxidase
MAC-IP: Membrane Attack Complex–Inhibitory Protein
MDA5: Melanoma Differentiation–Associated Protein 5
MDSC: Myeloid-Derived Suppressor Cell
miRNA: MicroRNA
mRNA: Messenger RNA
MSLN: Mesothelin
MSNs: Mesoporous Silica Nanoparticles
MSC: Mesenchymal Stem Cell
mtDNA/mtRNA: Mitochondrial DNA / Mitochondrial RNA
MVBs: Multivesicular Bodies
MYC: v-myc Avian Myelocytomatosis Viral Oncogene
NK: Natural Killer Cell
NTA: Nanoparticle Tracking Analysis
ORR: Overall Response Rate
PBS: Phosphate-Buffered Saline
PD-1/PD-L1: Programmed Death 1 Programmed Death-Ligand 1
PEG: Polyethylene Glycol
PiRNA: PIWI-Interacting RNA
PTX: Paclitaxel
RAB: Ras-Related Proteins in Vesicle Trafficking
RIG-I: Retinoic Acid–Inducible Gene I
RNA-seq: RNA Sequencing
RN7SL1: 7SL1 Noncoding RNA Used as Immune Adjuvant
rRNA: Ribosomal RNA
scFv: Single-Chain Variable Fragment
SEC: Size-Exclusion Chromatography
sgRNA: Single Guide RNA
siRNA: Small Interfering RNA
snRNA/snoRNA: Small Nuclear RNA / Small Nucleolar RNA
SPAAC: Strain-Promoted Alkyne–Azide Cycloaddition
SRC: Proto-Oncogene Tyrosine-Protein Kinase Src
SUMOylation: Small Ubiquitin-like Modifier Conjugation
TAA: Tumor-Associated Antigen
Tat-PEG-Lipids: Tat Peptide–Conjugated PEGylated Lipids
TCR: T Cell Receptor
TDE/TDEs: Tumor-Derived Exosome(s)
TFF: Tangential Flow Filtration
TGF-β: Transforming Growth Factor Beta
TIL: Tumor-Infiltrating Lymphocyte
TLRs: Toll-Like Receptors
TME: Tumor Microenvironment
Treg: Regulatory T cell
TSG101: Tumor Susceptibility Gene 101
tRNA/vtRNA/YRNA: Transfer RNA / Vault RNA / Y RNA
V-ATPase: Vacuolar-Type H+ ATPase
VEGF: Vascular Endothelial Growth Factor
VLP: Virus-Like Particle
VPS4: Vacuolar Protein Sorting 4

## References

[1] Johnstone RM, Adam M, Hammond JR, Orr L, Turbide C. Vesicle formation during reticulocyte maturation. Association of plasma membrane activities with released vesicles (exosomes). J Biol Chem. 1987;262(19):9412–20. 10.1016/S0021-9258(18)48095-7. 3597417

[2] Kalluri R, LeBleu VS. The biology, function, and biomedical applications of exosomes. Science. 2020;367(6478):eaau6977. 10.1126/science.aau6977. 32029601 10.1126/science.aau6977 PMC7717626

[3] Pegtel DM, Gould SJ. Exosomes. Annu Rev Biochem. 2019;88:487–514. 10.1146/annurev-biochem-013118-111902. 31220978 10.1146/annurev-biochem-013118-111902

[4] Wang Y, Xiao T, Zhao C, Li G. The regulation of exosome generation and function in physiological and pathological processes. Int J Mol Sci. 2023;25(1):255. 10.3390/ijms25010255. 38203424 10.3390/ijms25010255 PMC10779122

[5] Zhang Y, Liu Y, Liu H, Tang WH. Exosomes: biogenesis, biologic function and clinical potential. Cell Biosci. 2019;9(1):19. 10.1186/s13578-019-0282-2. 30815248 10.1186/s13578-019-0282-2 PMC6377728

[6] Skog J, Würdinger T, van Rijn S, Meijer DH, Gainche L, Sena-Esteves M, et al. Glioblastoma microvesicles transport RNA and proteins that promote tumour growth and provide diagnostic biomarkers. Nat Cell Biol. 2008;10(12):1470–6. 10.1038/ncb1800. 19011622 10.1038/ncb1800 PMC3423894

[7] Théry C, Witwer KW, Aikawa E, Alcaraz MJ, Anderson JD, Andriantsitohaina R, et al. Minimal information for studies of extracellular vesicles 2018 (MISEV2018): a position statement of the International Society for Extracellular Vesicles and update of the MISEV2014 guidelines. J Extracell Vesicles. 2018;7(1):1535750. 10.1080/20013078.2018.1535750. 30637094 10.1080/20013078.2018.1535750 PMC6322352

[8] Salman DM, Mohammad TAM. Leukemia cancer cells and immune cells derived-exosomes: possible roles in leukemia progression and therapy. Cell Biochem Funct. 2024;42(2):e3960. 10.1002/cbf.3960. 38424731 10.1002/cbf.3960

[9] Zemanek T, Danisovic L, Nicodemou A. Exosomes and solid cancer therapy: where are we now? Med Oncol. 2025;42(3):77. 10.1007/s12032-025-02626-3. 39961904 10.1007/s12032-025-02626-3 PMC11832697

[10] Zhang SH, Peng LL, Chen YF, Xu Y, Moradi V. Focusing on exosomes to overcome the existing bottlenecks of CAR-T cell therapy. Inflamm Regen. 2024;44(1):45. 10.1186/s41232-024-00358-x. 39490997 10.1186/s41232-024-00358-x PMC11533312

[11] Fu W, Lei C, Liu S, Cui Y, Wang C, Qian K, et al. CAR exosomes derived from effector CAR-T cells have potent antitumour effects and low toxicity. Nat Commun. 2019;10(1):4355. 10.1038/s41467-019-12321-3. 31554797 10.1038/s41467-019-12321-3 PMC6761190

[12] Xu Q, Zhang Z, Zhao L, Qin Y, Cai H, Geng Z, et al. Tropism-facilitated delivery of CRISPR/Cas9 system with chimeric antigen receptor-extracellular vesicles against B-cell malignancies. J Control Release. 2020;326:455–67. 10.1016/j.jconrel.2020.07.033. 32711027 10.1016/j.jconrel.2020.07.033

[13] Sunzi. The Art of War. Translated by Ralph D. Sawyer. Boulder: Westview Press; 1994. 375 p.

[14] Herrmann IK, Wood MJA, Fuhrmann G. Extracellular vesicles as a next-generation drug delivery platform. Nat Nanotechnol. 2021;16(7):748–59. 10.1038/s41565-021-00931-2. 34211166 10.1038/s41565-021-00931-2

[15] Woith E, Fuhrmann G, Melzig MF. Extracellular Vesicles-Connecting Kingdoms. Int J Mol Sci. 2019;20(22):5695. 10.3390/ijms20225695. 31739393 10.3390/ijms20225695 PMC6888613

[16] Li M, Liao L, Tian W. Extracellular vesicles derived from apoptotic cells: an essential link between death and regeneration. Front Cell Dev Biol. 2020;8:573511. 10.3389/fcell.2020.573511. 33134295 10.3389/fcell.2020.573511 PMC7561711

[17] Wang Y, Khan H, Zhou C, Liao X, Tang P, Song P, et al. Apoptotic cell-derived micro/nanosized extracellular vesicles in tissue regeneration. Nanotechnology Rev. 2022;11(1):957–72. 10.1515/ntrev-2022-0052.

[18] Jeppesen DK, Zhang Q, Coffey RJ. Extracellular vesicles and nanoparticles at a glance. J Cell Sci. 2024;137(23):jcs260201. 10.1242/jcs.260201. 39641198 10.1242/jcs.260201 PMC11658686

[19] Yáñez-Mó M, Siljander PR, Andreu Z, Zavec AB, Borràs FE, Buzas EI, et al. Biological properties of extracellular vesicles and their physiological functions. J Extracell Vesicles. 2015;4:27066. 10.3402/jev.v4.27066. 25979354 10.3402/jev.v4.27066 PMC4433489

[20] Abels ER, Breakefield XO. Introduction to extracellular vesicles: biogenesis, RNA cargo selection, content, release, and uptake. Cell Mol Neurobiol. 2016;36(3):301–12. 10.1007/s10571-016-0366-z. 27053351 10.1007/s10571-016-0366-z PMC5546313

[21] Jeppesen DK, Zhang Q, Franklin JL, Coffey RJ. Extracellular vesicles and nanoparticles: emerging complexities. Trends Cell Biol. 2023;33(8):667–81. 10.1016/j.tcb.2023.01.002. 36737375 10.1016/j.tcb.2023.01.002 PMC10363204

[22] Sheta M, Taha EA, Lu Y, Eguchi T. Extracellular vesicles: new classification and tumor immunosuppression. Biology. 2023;12(1):110. 10.3390/biology12010110. 36671802 10.3390/biology12010110 PMC9856004

[23] Di Bella MA. Overview and update on extracellular vesicles: considerations on exosomes and their application in modern medicine. Biology (Basel). 2022;11(6):804. 10.3390/biology11060804. 35741325 10.3390/biology11060804 PMC9220244

[24] Jin Y, Ma L, Zhang W, Yang W, Feng Q, Wang H. Extracellular signals regulate the biogenesis of extracellular vesicles. Biol Res. 2022;55(1):35. 10.1186/s40659-022-00405-2. 36435789 10.1186/s40659-022-00405-2 PMC9701380

[25] Lopez K, Lai SWT, Lopez Gonzalez EJ, Dávila RG, Shuck SC. Extracellular vesicles: a dive into their role in the tumor microenvironment and cancer progression. Front Cell Dev Biol. 2023;11:1154576. 10.3389/fcell.2023.1154576. 37025182 10.3389/fcell.2023.1154576 PMC10071009

[26] Saunderson SC, Dunn AC, Crocker PR, McLellan AD. CD169 mediates the capture of exosomes in spleen and lymph node. Blood. 2014;123(9):208–16. 10.1182/blood-2013-03-489732. 24255917 10.1182/blood-2013-03-489732 PMC3888287

[27] Martínez MC, Andriantsitohaina R. Extracellular vesicles in metabolic syndrome. Circ Res. 2017;120(10):1674–86. 10.1161/CIRCRESAHA.117.309419. 28495997 10.1161/CIRCRESAHA.117.309419

[28] Gurung S, Perocheau D, Touramanidou L, Baruteau J. The exosome journey: from biogenesis to uptake and intracellular signalling. Cell Commun Signal. 2021;19(23):47. 10.1186/s12964-021-00730-1. 33892745 10.1186/s12964-021-00730-1 PMC8063428

[29] da Costa VR, Araldi RP, Vigerelli H, D’Ámelio F, Mendes TB, Gonzaga V, et al. Exosomes in the tumor microenvironment: from biology to clinical applications. Cells. 2021;10(10):2617. 10.3390/cells10102617. 34685596 10.3390/cells10102617 PMC8533895

[30] Liu YJ, Wang C. A review of the regulatory mechanisms of extracellular vesicles-mediated intercellular communication. Cell Commun Signal. 2023;21(1):77. 10.1186/s12964-023-01103-6. 37055761 10.1186/s12964-023-01103-6 PMC10100201

[31] Raposo G, Stoorvogel W. Extracellular vesicles: exosomes, microvesicles, and friends. J Cell Biol. 2013;200(4):373–83. 10.1083/jcb.201211138. 23420871 10.1083/jcb.201211138 PMC3575529

[32] Fendl B, Eichhorn T, Weiss R, Tripisciano C, Spittler A, Fischer MB, et al. Differential interaction of Platelet-Derived extracellular vesicles with Circulating immune cells: roles of TAM Receptors, CD11b, and phosphatidylserine. Front Immunol. 2018;9:2797. 10.3389/fimmu.2018.02797. 30619243 10.3389/fimmu.2018.02797 PMC6297748

[33] Limongi T, Susa F, Dumontel B, Racca L, Perrone Donnorso M, Debellis D, et al. Extracellular vesicles tropism: a comparative study between passive innate tropism and the active engineered targeting capability of lymphocyte-derived EVs. Membranes (Basel). 2021;11(18):886. 10.3390/membranes11110886. 34832115 10.3390/membranes11110886 PMC8617986

[34] Ibrahim A, Marbán E. Exosomes: fundamental biology and roles in cardiovascular physiology. Annu Rev Physiol. 2016;78(1):67–83. 10.1146/annurev-physiol-021115-104929. 26667071 10.1146/annurev-physiol-021115-104929 PMC5425157

[35] Krylova SV, Feng D. The machinery of exosomes: biogenesis, release, and uptake. Int J Mol Sci. 2023;24(2):1337. 10.3390/ijms24021337. 36674857 10.3390/ijms24021337 PMC9865891

[36] Lee YJ, Shin KJ, Chae YC. Regulation of cargo selection in exosome biogenesis and its biomedical applications in cancer. Exp Mol Med. 2024;56(4):877–89. 10.1038/s12276-024-01209-y. 38580812 10.1038/s12276-024-01209-y PMC11059157

[37] Skotland T, Sandvig K, Llorente A. Lipids in exosomes: current knowledge and the way forward. Prog Lipid Res. 2017;66:30–41. 10.1016/j.plipres.2017.03.001. 28342835 10.1016/j.plipres.2017.03.001

[38] Lu J, Wu J, Tian J, Wang S. Role of t cell-derived exosomes in immunoregulation. Immunol Res. 2018;66(3):313–22. 10.1007/s12026-018-9000-0. 29804198 10.1007/s12026-018-9000-0

[39] Qiu Y, Yang Y, Yang R, Liu C, Hsu JM, Jiang Z, et al. Activated t cell-derived exosomal PD-1 attenuates PD-L1-induced immune dysfunction in triple-negative breast cancer. Oncogene. 2021;40(31):4992–5001. 10.1038/s41388-021-01896-1. 34172932 10.1038/s41388-021-01896-1 PMC8342306

[40] Porcelli L, Guida M, De Summa S, Di Fonte R, De Risi I, Garofoli M, et al. uPAR+ extracellular vesicles: a robust biomarker of resistance to checkpoint inhibitor immunotherapy in metastatic melanoma patients. J Immunother Cancer. 2021;9(5):e002372. 10.1136/jitc-2021-002372. 33972390 10.1136/jitc-2021-002372 PMC8112420

[41] Zhang H, Xie Y, Li W, Chibbar R, Xiong S, Xiang J. CD4(+) t cell-released exosomes inhibit CD8(+) cytotoxic t-lymphocyte responses and antitumor immunity. Cell Mol Immunol. 2011;8(1):23–30. 10.1038/cmi.2010.59. 21200381 10.1038/cmi.2010.59 PMC4002994

[42] Cai Z, Yang F, Yu L, Yu Z, Jiang L, Wang Q, et al. Activated T cell exosomes promote tumor invasion via Fas signaling pathway. J Immunol. 2012;188(12):5954–61. 10.4049/jimmunol.1103466. 22573809 10.4049/jimmunol.1103466

[43] Zhou Q, Wei S, Wang H, Li Y, Fan S, Cao Y, et al. T cell-derived exosomes in tumor immune modulation and immunotherapy. Front Immunol. 2023;14:1130033. 10.3389/fimmu.2023.1130033. 37153615 10.3389/fimmu.2023.1130033 PMC10157026

[44] Abrami L, Brandi L, Moayeri M, Brown MJ, Krantz BA, Leppla SH, et al. Hijacking multivesicular bodies enables long-term and exosome-mediated long-distance action of anthrax toxin. Cell Rep. 2013;5(4):986–96. 10.1016/j.celrep.2013.10.019. 24239351 10.1016/j.celrep.2013.10.019 PMC3866279

[45] Okoye IS, Coomes SM, Pelly VS, Czieso S, Papayannopoulos V, Tolmachova T, et al. MicroRNA-containing T-regulatory-cell-derived exosomes suppress pathogenic T helper 1 cells. Immunity. 2014;41(1):89–103. 10.1016/j.immuni.2014.05.019. 25035954 10.1016/j.immuni.2014.05.019 PMC4104030

[46] Theodoraki MN, Yerneni S, Gooding WE, Ohr J, Clump DA, Bauman JE, et al. Circulating exosomes measure responses to therapy in head and neck cancer patients treated with cetuximab, ipilimumab, and IMRT. Oncoimmunology. 2019;8(7):1593805. 10.1080/2162402X.2019.1593805. 31143513 10.1080/2162402X.2019.1593805 PMC6527269

[47] Tung SL, Boardman DA, Sen M, Letizia M, Peng Q, Cianci N, et al. Regulatory T cell-derived extracellular vesicles modify dendritic cell function. Sci Rep. 2018;8(1):6065. 10.1038/s41598-018-24531-8. 29666503 10.1038/s41598-018-24531-8 PMC5904112

[48] Mathieu M, Martin-Jaular L, Lavieu G, Théry C. Specificities of secretion and uptake of exosomes and other extracellular vesicles for cell-to-cell communication. Nat Cell Biol. 2019;21(1):9–17. 10.1038/s41556-018-0250-9. 30602770 10.1038/s41556-018-0250-9

[49] Kowal J, Arras G, Colombo M, Jouve M, Morath JP, Primdal-Bengtson B, et al. Proteomic comparison defines novel markers to characterize heterogeneous populations of extracellular vesicle subtypes. Proc Natl Acad Sci U S A. 2016;113(8):E968-77. 10.1073/pnas.1521230113. 26858453 10.1073/pnas.1521230113 PMC4776515

[50] Fordjour FK, Guo C, Ai Y, Daaboul GG, Gould SJ. A shared, stochastic pathway mediates exosome protein budding along plasma and endosome membranes. J Biol Chem. 2022;298(10):102394. 10.1016/j.jbc.2022.102394. 35988652 10.1016/j.jbc.2022.102394 PMC9512851

[51] Guo W, Cai Y, Liu X, Ji Y, Zhang C, Wang L, et al. Single-exosome profiling identifies ITGB3 + and ITGAM+ exosome subpopulations as promising early diagnostic biomarkers and therapeutic targets for colorectal cancer. Research. 2023;6:0041. 10.34133/research.0041. 37040507 10.34133/research.0041 PMC10076010

[52] Livshits MA, Khomyakova E, Evtushenko EG, Lazarev VN, Kulemin NA, Semina SE, et al. Isolation of exosomes by differential centrifugation: theoretical analysis of a commonly used protocol. Sci Rep. 2015;5:17319. 10.1038/srep17319. 26616523 10.1038/srep17319 PMC4663484

[53] Zhang Y, Bi J, Huang J, Tang Y, Du S, Li P. Exosome: a review of its classification, isolation techniques, storage, diagnostic and targeted therapy applications. Int J Nanomedicine. 2020;15:6917–34. 10.2147/IJN.S264498. 33061359 10.2147/IJN.S264498 PMC7519827

[54] Oh DK, Hyun CK, Kim JH, Park YH. Production of penicillin in a fluidized-bed bioreactor: control of cell growth and penicillin production by phosphate limitation. Biotechnol Bioeng. 1988;32(4):569–73. 10.1002/bit.260320421. 18587756 10.1002/bit.260320421

[55] Vergauwen G, Dhondt B, Van Deun J, De Smedt E, Berx G, Timmerman E, et al. Confounding factors of ultrafiltration and protein analysis in extracellular vesicle research. Sci Rep. 2017;7(1):2704. 10.1038/s41598-017-02599-y. 28577337 10.1038/s41598-017-02599-y PMC5457435

[56] van der Böing AN, Grootemaat AE, Coumans FA, Sturk A, Nieuwland R. Single-step isolation of extracellular vesicles by size-exclusion chromatography. J Extracell Vesicles. 2014;3. 10.3402/jev.v3.23430. 10.3402/jev.v3.23430 PMC4159761 25279113

[57] Tschuschke M, Kocherova I, Bryja A, Mozdziak P, Angelova Volponi A, Janowicz K, et al. Inclusion biogenesis, methods of isolation and clinical application of human cellular exosomes. J Clin Med. 2020;9(2):436. 10.3390/jcm9020436. 32041096 10.3390/jcm9020436 PMC7074492

[58] Sidhom K, Obi PO, Saleem A. A review of exosomal isolation methods: is size exclusion chromatography the best option? Int J Mol Sci. 2020;21(18):6466. 10.3390/ijms21186466. 32899828 10.3390/ijms21186466 PMC7556044

[59] Gurunathan S, Kang MH, Jeyaraj M, Qasim M, Kim JH. Review of the isolation, characterization, biological function, and multifarious therapeutic approaches of exosomes. Cells. 2019;8(4):307. 10.3390/cells8040307. 30987213 10.3390/cells8040307 PMC6523673

[60] Alzhrani GN, Alanazi ST, Alsharif SY, Albalawi AM, Alsharif AA, Abdel-Maksoud MS, et al. Exosomes: isolation, characterization, and biomedical applications. Cell Biol Int. 2021;45(9):1807–31. 10.1002/cbin.11620. 33913604 10.1002/cbin.11620

[61] Mishra A, Bharti PS, Rani N, Nikolajeff F, Kumar S. A tale of exosomes and their implication in cancer. Biochimica et Biophysica Acta (BBA) - Reviews on Cancer. 2023;1878(4):188908. 10.1016/j.bbcan.2023.188908. 37172650 10.1016/j.bbcan.2023.188908

[62] Tiwari S, Kumar V, Randhawa S, Verma SK. Preparation and characterization of extracellular vesicles. Am J Reprod Immunol. 2021;85(2):e13367. 10.1111/aji.13367. 33118232 10.1111/aji.13367

[63] Yuan F, Li YM, Wang Z. Preserving extracellular vesicles for biomedical applications: consideration of storage stability before and after isolation. Drug Deliv. 2021;28(1):1501–9. 10.1080/10717544.2021.1951896. 34259095 10.1080/10717544.2021.1951896 PMC8281093

[64] Witwer KW, Buzás EI, Bemis LT, Bora A, Lässer C, Lötvall J, et al. Standardization of sample collection, isolation and analysis methods in extracellular vesicle research. J Extracell Vesicles. 2013. 10.3402/jev.v2i0.20360. 24009894 10.3402/jev.v2i0.20360 PMC3760646

[65] Wu JY, Li YJ, Hu XB, Huang S, Xiang DX. Preservation of small extracellular vesicles for functional analysis and therapeutic applications: a comparative evaluation of storage conditions. Drug Deliv. 2021;28(1):162–70. 10.1080/10717544.2020.1869866. 33427518 10.1080/10717544.2020.1869866 PMC7808382

[66] Jeyaram A, Jay SM. Preservation and storage stability of extracellular vesicles for therapeutic applications. AAPS J. 2017;20(1):1. 10.1208/s12248-017-0160-y. 29181730 10.1208/s12248-017-0160-y PMC6582961

[67] Welsh JA, Goberdhan DCI, O’Driscoll L, Buzas EI, Blenkiron C, Bussolati B, et al. Minimal information for studies of extracellular vesicles (MISEV2023): from basic to advanced approaches. J Extracell Vesicles. 2024;13(2):e12404. 10.1002/jev2.12404. 38326288 10.1002/jev2.12404 PMC10850029

[68] Luo C, Xin H, Zhou Z, Hu Z, Sun R, Yao N, et al. Tumor-derived exosomes induce immunosuppressive macrophages to foster intrahepatic cholangiocarcinoma progression. Hepatology. 2022;76(4):982–99. 10.1002/hep.32387. 35106794 10.1002/hep.32387

[69] Raimondo S, Saieva L, Corrado C, Fontana S, Flugy A, Rizzo A, et al. Chronic myeloid leukemia-derived exosomes promote tumor growth through an autocrine mechanism. Cell Commun Signal. 2015;13:8. 10.1186/s12964-015-0086-x. 25644060 10.1186/s12964-015-0086-x PMC4320527

[70] Qu JL, Qu XJ, Zhao MF, Teng YE, Zhang Y, Hou KZ, et al. Gastric cancer exosomes promote tumour cell proliferation through PI3K/Akt and MAPK/ERK activation. Dig Liver Dis. 2009;41(12):875–80. 10.1016/j.dld.2009.04.006. 19473897 10.1016/j.dld.2009.04.006

[71] Yang L, Wu XH, Wang D, Luo CL, Chen LX. Bladder cancer cell-derived exosomes inhibit tumor cell apoptosis and induce cell proliferation in vitro. Mol Med Rep. 2013;8(4):1272–8. 10.3892/mmr.2013.1634. 23969721 10.3892/mmr.2013.1634

[72] Matsumoto A, Takahashi Y, Nishikawa M, Sano K, Morishita M, Charoenviriyakul C, et al. Accelerated growth of B16BL6 tumor in mice through efficient uptake of their own exosomes by B16BL6 cells. Cancer Sci. 2017;108(9):1803–10. 10.1111/cas.13310. 28667694 10.1111/cas.13310 PMC5581513

[73] Ramteke A, Ting H, Agarwal C, Mateen S, Somasagara R, Hussain A, et al. Exosomes secreted under hypoxia enhance invasiveness and stemness of prostate cancer cells by targeting adherens junction molecules. Mol Carcinog. 2015;54(7):554–65. 10.1002/mc.22124. 24347249 10.1002/mc.22124 PMC4706761

[74] Arneth B. Tumor microenvironment. Med (Kaunas). 2019;56(1):15. 10.3390/medicina56010015. 10.3390/medicina56010015 PMC7023392 31906017

[75] Xiao Y, Yu D. Tumor microenvironment as a therapeutic target in cancer. Pharmacol Ther. 2021;221:107753. 10.1016/j.pharmthera.2020.107753. 33259885 10.1016/j.pharmthera.2020.107753 PMC8084948

[76] Kamerkar S, Leng C, Burenkova O, Jang SC, McCoy C, Zhang K, et al. Exosome-mediated genetic reprogramming of tumor-associated macrophages by exoASO-STAT6 leads to potent monotherapy antitumor activity. Sci Adv. 2022;8(7):eabj7002. 10.1126/sciadv.abj7002. 35179953 10.1126/sciadv.abj7002 PMC8856615

[77] Mondal SK, Haas D, Han J, Whiteside TL. Small EV in plasma of triple negative breast cancer patients induce intrinsic apoptosis in activated T cells. Commun Biol. 2023;6(1):815. 10.1038/s42003-023-05169-3. 37542121 10.1038/s42003-023-05169-3 PMC10403597

[78] Chatterjee S, Chatterjee A, Jana S, Dey S, Roy H, Das MK, et al. Transforming growth factor beta orchestrates PD-L1 enrichment in tumor-derived exosomes and mediates CD8 T-cell dysfunction regulating early phosphorylation of TCR signalome in breast cancer. Carcinogenesis. 2021;42(1):38–47. 10.1093/carcin/bgaa092. 32832992 10.1093/carcin/bgaa092

[79] Whiteside TL. Tumor-derived exosomes and antitumor immunity. J Immunol. 2024;213(7):923–31. 10.4049/jimmunol.2400335. 39284119 10.4049/jimmunol.2400335 PMC11951267

[80] Vulpis E, Loconte L, Peri A, Molfetta R, Caracciolo G, Masuelli L, et al. Impact on NK cell functions of acute versus chronic exposure to extracellular vesicle-associated MICA: dual role in cancer immunosurveillance. J Extracell Vesicles. 2022;11(1):e12176. 10.1002/jev2.12176. 34973063 10.1002/jev2.12176 PMC8720178

[81] Yu S, Sha H, Qin X, Chen Y, Li X, Shi M, et al. EGFR E746-A750 deletion in lung cancer represses antitumor immunity through the exosome-mediated inhibition of dendritic cells. Oncogene. 2020;39(13):2643–57. 10.1038/s41388-020-1182-y. 32001818 10.1038/s41388-020-1182-y

[82] Huyan T, Gao L, Gao N, Wang C, Guo W, Zhou X, et al. miR-221-5p and miR-186-5p are the critical bladder cancer derived exosomal miRNAs in natural killer cell dysfunction. Int J Mol Sci. 2022;23(23):15177. 10.3390/ijms232315177. 36499501 10.3390/ijms232315177 PMC9740765

[83] Schroeder JC, Puntigam L, Hofmann L, Jeske SS, Beccard IJ, Doescher J, et al. Circulating exosomes inhibit B cell proliferation and activity. Cancers (Basel). 2020;12(8):2110. 10.3390/cancers12082110. 32751214 10.3390/cancers12082110 PMC7464446

[84] Zhou C, Wei W, Ma J, Yang Y, Liang L, Zhang Y, et al. Cancer-secreted exosomal miR-1468-5p promotes tumor immune escape via the immunosuppressive reprogramming of lymphatic vessels. Mol Ther. 2021;29(4):1512–28. 10.1016/j.ymthe.2020.12.034. 33388421 10.1016/j.ymthe.2020.12.034 PMC8058488

[85] Baig MS, Roy A, Rajpoot S, Liu D, Savai R, Banerjee S, et al. Tumor-derived exosomes in the regulation of macrophage polarization. Inflamm Res. 2020;69(5):435–51. 10.1007/s00011-020-01318-0. 32162012 10.1007/s00011-020-01318-0

[86] Huang YK, Wang M, Sun Y, Di Costanzo N, Mitchell C, Achuthan A, et al. Macrophage spatial heterogeneity in gastric cancer defined by multiplex immunohistochemistry. Nat Commun. 2019;10(1):3928. 10.1038/s41467-019-11788-4. 31477692 10.1038/s41467-019-11788-4 PMC6718690

[87] Chen S, Sun J, Zhou H, Lei H, Zang D, Chen J. New roles of tumor-derived exosomes in tumor microenvironment. Chin J Cancer Res. 2024;36(2):151–66. 10.21147/j.issn.1000-9604.2024.02.05. 38751437 10.21147/j.issn.1000-9604.2024.02.05 PMC11090792

[88] Yang C, Dou R, Wei C, Liu K, Shi D, Zhang C, et al. Tumor-derived exosomal microRNA-106b-5p activates EMT-cancer cell and M2-subtype TAM interaction to facilitate CRC metastasis. Mol Ther. 2021;29(6):2088–107. 10.1016/j.ymthe.2021.02.006. 33571679 10.1016/j.ymthe.2021.02.006 PMC8178444

[89] Rao X, Zhou X, Wang G, Jie X, Xing B, Xu Y, et al. NLRP6 is required for cancer-derived exosome-modified macrophage M2 polarization and promotes metastasis in small cell lung cancer. Cell Death Dis. 2022;13(10):891. 10.1038/s41419-022-05336-0. 36270983 10.1038/s41419-022-05336-0 PMC9587220

[90] Park JE, Dutta B, Tse SW, Gupta N, Tan CF, Low JK, et al. Hypoxia-induced tumor exosomes promote M2-like macrophage polarization of infiltrating myeloid cells and microRNA-mediated metabolic shift. Oncogene. 2019;38(26):5158–73. 10.1038/s41388-019-0782-x. 30872795 10.1038/s41388-019-0782-x

[91] Ye Z, Li G, Lei J. Influencing immunity: role of extracellular vesicles in tumor immune checkpoint dynamics. Exp Mol Med. 2024;56(11):2365–81. 10.1038/s12276-024-01340-w. 39528800 10.1038/s12276-024-01340-w PMC11612210

[92] Mendes M, Monteiro AC, Neto E, Barrias CC, Sobrinho-Simões MA, Duarte D, et al. Transforming the niche: the emerging role of extracellular vesicles in acute myeloid leukaemia progression. Int J Mol Sci. 2024;25(8):4430. 10.3390/ijms25084430. 38674015 10.3390/ijms25084430 PMC11050723

[93] Li F, Zhan L, Dong Q, Wang Q, Wang Y, Li X, et al. Tumor-derived exosome-educated hepatic stellate cells regulate lactate metabolism of hypoxic colorectal tumor cells via the IL-6/STAT3 pathway to confer drug resistance. Onco Targets Ther. 2020;13:7851–64. 10.2147/OTT.S253485. 32821126 10.2147/OTT.S253485 PMC7423356

[94] Ahn M, Mun JG, Han Y, Seo JH. Cancer cell-derived extracellular vesicles: a potential target for overcoming tumor immunotherapy resistance and immune evasion strategies. Front Immunol. 2025;16:1601266. 10.3389/fimmu.2025.1601266. 40574844 10.3389/fimmu.2025.1601266 PMC12198139

[95] Yang X, Zhang Y, Zhang Y, Zhang S, Qiu L, Zhuang Z, et al. The key role of exosomes on the pre-metastatic niche formation in tumors. Front Mol Biosci. 2021;8:703640. 10.3389/fmolb.2021.703640. 34595207 10.3389/fmolb.2021.703640 PMC8476876

[96] Fabbri M, Paone A, Calore F, Galli R, Gaudio E, Santhanam R, et al. MicroRNAs bind to toll-like receptors to induce prometastatic inflammatory response. Proc Natl Acad Sci U S A. 2012;109(31):E2110-6. 10.1073/pnas.1209414109. 22753494 10.1073/pnas.1209414109 PMC3412003

[97] Gu P, Sun M, Li L, Yang Y, Jiang Z, Ge Y, et al. Breast tumor-derived exosomal MicroRNA-200b-3p promotes specific organ metastasis through regulating CCL2 expression in lung epithelial cells. Front Cell Dev Biol. 2021;9:657158. 10.3389/fcell.2021.657158. 34249913 10.3389/fcell.2021.657158 PMC8264457

[98] Treps L, Perret R, Edmond S, Ricard D, Gavard J. Glioblastoma stem-like cells secrete the pro-angiogenic VEGF-A factor in extracellular vesicles. J Extracell Vesicles. 2017;6(1):1359479. 10.1080/20013078.2017.1359479. 28815003 10.1080/20013078.2017.1359479 PMC5549846

[99] Yokota Y, Noda T, Okumura Y, Kobayashi S, Iwagami Y, Yamada D, et al. Serum exosomal miR-638 is a prognostic marker of HCC via downregulation of VE-cadherin and ZO-1 of endothelial cells. Cancer Sci. 2021;112(3):1275–88. 10.1111/cas.14807. 33426736 10.1111/cas.14807 PMC7935782

[100] Biagioni A, Laurenzana A, Menicacci B, Peppicelli S, Andreucci E, Bianchini F, et al. uPAR-expressing melanoma exosomes promote angiogenesis by VE-cadherin, EGFR and uPAR overexpression and rise of ERK1,2 signaling in endothelial cells. Cell Mol Life Sci. 2021;78(6):3057–72. 10.1007/s00018-020-03707-4. 33237352 10.1007/s00018-020-03707-4 PMC8004497

[101] Tadokoro H, Umezu T, Ohyashiki K, Hirano T, Ohyashiki JH. Exosomes derived from hypoxic leukemia cells enhance tube formation in endothelial cells. J Biol Chem. 2013;288(48):34343–51. 10.1074/jbc.M113.480822. 24133215 10.1074/jbc.M113.480822 PMC3843049

[102] Hsu YL, Hung JY, Chang WA, Lin YS, Pan YC, Tsai PH, et al. Hypoxic lung cancer-secreted exosomal miR-23a increased angiogenesis and vascular permeability by targeting prolyl hydroxylase and tight junction protein ZO-1. Oncogene. 2017;36(34):4929–42. 10.1038/onc.2017.105. 28436951 10.1038/onc.2017.105

[103] Chen C, Luo Y, He W, Zhao Y, Kong Y, Liu H, et al. Exosomal long noncoding RNA LNMAT2 promotes lymphatic metastasis in bladder cancer. J Clin Invest. 2020;130(1):404–21. 10.1172/JCI130892. 31593555 10.1172/JCI130892 PMC6934220

[104] Zhou CF, Ma J, Huang L, Yi HY, Zhang YM, Wu XG, et al. Cervical squamous cell carcinoma-secreted exosomal miR-221-3p promotes lymphangiogenesis and lymphatic metastasis by targeting VASH1. Oncogene. 2019;38(8):1256–68. 10.1038/s41388-018-0511-x. 30254211 10.1038/s41388-018-0511-x PMC6363643

[105] Pucci F, Garris C, Lai CP, Newton A, Pfirschke C, Engblom C, et al. SCS macrophages suppress melanoma by restricting tumor-derived vesicle-B cell interactions. Science. 2016;352(6282):242–6. 10.1126/science.aaf1328. 26989197 10.1126/science.aaf1328 PMC4960636

[106] Li M, Lu Y, Xu Y, Wang J, Zhang C, Du Y, et al. Horizontal transfer of exosomal CXCR4 promotes murine hepatocarcinoma cell migration, invasion and lymphangiogenesis. Gene. 2018;676:101–9. 10.1016/j.gene.2018.07.018. 30010038 10.1016/j.gene.2018.07.018

[107] Hoshino A, Costa-Silva B, Shen TL, Rodrigues G, Hashimoto A, Tesic Mark M, et al. Tumour exosome integrins determine organotropic metastasis. Nature. 2015;527(7578):329–35. 10.1038/nature15756. 26524530 10.1038/nature15756 PMC4788391

[108] Chen GY, Cheng JC, Chen YF, Yang JC, Hsu FM. Circulating exosomal integrin β3 is associated with intracranial failure and survival in lung cancer patients receiving cranial irradiation for brain metastases: a prospective observational study. Cancers (Basel). 2021;13(3):380. 10.3390/cancers13030380. 33498505 10.3390/cancers13030380 PMC7864205

[109] Zhang H, Deng T, Liu R, Bai M, Zhou L, Wang X, et al. Exosome-delivered EGFR regulates liver microenvironment to promote gastric cancer liver metastasis. Nat Commun. 2017;8(1):15016. 10.1038/ncomms15016. 28393839 10.1038/ncomms15016 PMC5394240

[110] Costa-Silva B, Aiello NM, Ocean AJ, Singh S, Zhang H, Thakur BK, et al. Pancreatic cancer exosomes initiate pre-metastatic niche formation in the liver. Nat Cell Biol. 2015;17(6):816–26. 10.1038/ncb3169. 25985394 10.1038/ncb3169 PMC5769922

[111] Wang L, Yang G, Zhao D, Wang J, Bai Y, Peng Q, et al. CD103-positive CSC exosome promotes EMT of clear cell renal cell carcinoma: role of remote MiR-19b-3p. Mol Cancer. 2019;18(1):86. 10.1186/s12943-019-0997-z. 30975145 10.1186/s12943-019-0997-z PMC6458839

[112] Qi M, Xia Y, Wu Y, Zhang Z, Wang X, Lu L, et al. Lin28B-high breast cancer cells promote immune suppression in the lung pre-metastatic niche via exosomes and support cancer progression. Nat Commun. 2022;13(1):897. 10.1038/s41467-022-28438-x. 35173168 10.1038/s41467-022-28438-x PMC8850492

[113] Kuang L, Wu L, Li Y. Extracellular vesicles in tumor immunity: mechanisms and novel insights. Mol Cancer. 2025;24(1):45. 10.1186/s12943-025-02233-w. 39953480 10.1186/s12943-025-02233-w PMC11829561

[114] Ploeg EM, Ke X, Britsch I, Hendriks MAJM, Van der Zant FA, Kruijff S, et al. Bispecific antibody CD73xEpCAM selectively inhibits the adenosine-mediated immunosuppressive activity of carcinoma-derived extracellular vesicles. Cancer Lett. 2021;521:109–18. 10.1016/j.canlet.2021.08.037. 34464670 10.1016/j.canlet.2021.08.037

[115] Wang X, Liu Y, Jiang Y, Li Q. Tumor-derived exosomes as promising tools for cancer diagnosis and therapy. Front Pharmacol. 2025;16:1596217. 10.3389/fphar.2025.1596217. 40444049 10.3389/fphar.2025.1596217 PMC12119533

[116] Wu X, Kang M, Wang D, Zhu M, Hu Y, Zhang Y, et al. Heparan sulfate analogues regulate tumor-derived exosome formation that attenuates exosome functions in tumor processes. Int J Biol Macromol. 2021;187:481–91. 10.1016/j.ijbiomac.2021.07.110. 34298051 10.1016/j.ijbiomac.2021.07.110

[117] Peng Y, Zhao M, Hu Y, Guo H, Zhang Y, Huang Y, et al. Blockade of exosome generation by GW4869 inhibits the education of M2 macrophages in prostate cancer. BMC Immunol. 2022;23(1):37. 10.1186/s12865-022-00514-3. 35941539 10.1186/s12865-022-00514-3 PMC9361607

[118] Kim JH, Lee CH, Baek MC. Dissecting exosome inhibitors: therapeutic insights into small-molecule chemicals against cancer. Exp Mol Med. 2022;54(11):1833–43. 10.1038/s12276-022-00898-7. 36446847 10.1038/s12276-022-00898-7 PMC9707221

[119] Koch R, Aung T, Vogel D, Chapuy B, Wenzel D, Becker S, et al. Nuclear trapping through inhibition of exosomal export by indomethacin increases cytostatic efficacy of doxorubicin and pixantrone. Clin Cancer Res. 2016;22(2):395–404. 10.1158/1078-0432.CCR-15-0577. 26369630 10.1158/1078-0432.CCR-15-0577

[120] Sasabe E, Tomomura A, Liu H, Sento S, Kitamura N, Yamamoto T. Epidermal growth factor/epidermal growth factor receptor signaling blockage inhibits tumor cell-derived exosome uptake by oral squamous cell carcinoma through macropinocytosis. Cancer Sci. 2022;113(2):609–21. 10.1111/cas.15225. 34874595 10.1111/cas.15225 PMC8819298

[121] Datta A, Kim H, Lal M, McGee L, Johnson A, Moustafa AA, et al. Manumycin A suppresses exosome biogenesis and secretion via targeted inhibition of Ras/Raf/ERK1/2 signaling and hnRNP H1 in castration-resistant prostate cancer cells. Cancer Lett. 2017;408:73–81. 10.1016/j.canlet.2017.08.020. 28844715 10.1016/j.canlet.2017.08.020 PMC5628151

[122] Roseblade A, Luk F, Ung A, Bebawy M. Targeting microparticle biogenesis: a novel approach to the circumvention of cancer multidrug resistance. Curr Cancer Drug Targets. 2015;15(3):205–14. 10.2174/1568009615666150225121508. 25714701 10.2174/1568009615666150225121508

[123] Li B, Antonyak MA, Zhang J, Cerione RA. RhoA triggers a specific signaling pathway that generates transforming microvesicles in cancer cells. Oncogene. 2012;31(8):4740–9. 10.1038/onc.2011.636. 22266864 10.1038/onc.2011.636 PMC3607381

[124] Garcia M, Hoffer L, Leblanc R, Benmansour F, Feracci M, Derviaux C, et al. Fragment-based drug design targeting Syntenin PDZ2 domain involved in Exosomal release and tumour spread. Eur J Med Chem. 2021;223:113601. 10.1016/j.ejmech.2021.113601. 34153575 10.1016/j.ejmech.2021.113601

[125] Ye Z, Xiong Y, Peng W, Wei W, Huang L, Yue J, et al. Manipulation of PD-L1 endosomal trafficking promotes anticancer immunity. Adv Sci. 2023;10(6):e2206411. 10.1002/advs.202206411. 10.1002/advs.202206411 PMC9951344 36567273

[126] Chen HL, Luo YP, Lin MW, Peng XX, Liu ML, Wang YC, et al. Serum exosomal miR-16-5p functions as a tumor inhibitor and a new biomarker for PD-L1 inhibitor-dependent immunotherapy in lung adenocarcinoma by regulating PD-L1 expression. Cancer Med. 2022;11(13):2627–43. 10.1002/cam4.4638. 35347894 10.1002/cam4.4638 PMC9249988

[127] Sella A, Kilbourn R, Amato R, Bui C, Zukiwski AA, Ellerhorst J, et al. Phase II study of ketoconazole combined with weekly doxorubicin in patients with androgen-independent prostate cancer. J Clin Oncol. 1994;12(4):683–8. 10.1200/JCO.1994.12.4.683. 7512126 10.1200/JCO.1994.12.4.683

[128] Salarpour S, Forootanfar H, Pournamdari M, Ahmadi-Zeidabadi M, Esmaeeli M, Pardakhty A. Paclitaxel incorporated exosomes derived from glioblastoma cells: comparative study of two loading techniques. Daru. 2019;27(2):533–9. 10.1007/s40199-019-00280-5. 31317441 10.1007/s40199-019-00280-5 PMC6895332

[129] Saari H, Lázaro-Ibáñez E, Viitala T, Vuorimaa-Laukkanen E, Siljander P, Yliperttula M. Microvesicle- and exosome-mediated drug delivery enhances the cytotoxicity of Paclitaxel in autologous prostate cancer cells. J Control Release. 2015;220(Pt B):727–37. 10.1016/j.jconrel.2015.09.031. 26390807 10.1016/j.jconrel.2015.09.031

[130] Cui Z, Ruan Z, Zeng J, Sun J, Ye W, Xu W, et al. Lung-specific exosomes for co-delivery of CD47 blockade and cisplatin for the treatment of non-small cell lung cancer. Thorac Cancer. 2022;13(19):2723–31. 10.1111/1759-7714.14606. 36054073 10.1111/1759-7714.14606 PMC9527158

[131] Jiang Y, Xu X, Fan D, Liu P, Zhou M, Cheng M, et al. Advancing Tumor-Targeted Chemo-Immunotherapy: development of the CAR-M-derived Exosome-Drug conjugate. J Med Chem. 2024;67(16):13959–74. 10.1021/acs.jmedchem.4c00753. 39041307 10.1021/acs.jmedchem.4c00753

[132] Antimisiaris SG, Mourtas S, Marazioti A. Exosomes and exosome-inspired vesicles for targeted drug delivery. Pharmaceutics. 2018;10(4):218. 10.3390/pharmaceutics10040218. 30404188 10.3390/pharmaceutics10040218 PMC6321407

[133] Niu H, Zhao P, Sun W. Biomaterials for chimeric antigen receptor T cell engineering. Acta Biomater. 2023;166:1–13. 10.1016/j.actbio.2023.04.043. 37137403 10.1016/j.actbio.2023.04.043

[134] Ruan S, Huang Y, He M, Gao H. Advanced biomaterials for cell-specific modulation and restore of cancer immunotherapy. Adv Sci. 2022;9(16):e2200027. 10.1002/advs.202200027. 10.1002/advs.202200027 PMC9165523 35343112

[135] Shao J, Zaro J, Shen Y. Advances in exosome-based drug delivery and tumor targeting: from tissue distribution to intracellular fate. Int J Nanomed. 2020;15:9355–71. 10.2147/IJN.S281890. 10.2147/IJN.S281890 PMC7700079 33262592

[136] Song LL, Tang YP, Qu YQ, Yun YX, Zhang RL, Wang CR, et al. Exosomal delivery of rapamycin modulates blood-brain barrier penetration and VEGF axis in glioblastoma. J Control Release. 2025;381:113605. 10.1016/j.jconrel.2025.113605. 40058500 10.1016/j.jconrel.2025.113605

[137] Alvarez-Erviti L, Seow Y, Yin H, Betts C, Lakhal S, Wood MJ. Delivery of siRNA to the mouse brain by systemic injection of targeted exosomes. Nat Biotechnol. 2011;29(4):341–5. 10.1038/nbt.1807. 21423189 10.1038/nbt.1807

[138] Schuster SJ, Svoboda J, Chong EA, Nasta SD, Mato AR, Anak Ö, et al. Chimeric antigen receptor T cells in refractory B-Cell lymphomas. N Engl J Med. 2017;377(26):2545–54. 10.1056/NEJMoa1708566. 29226764 10.1056/NEJMoa1708566 PMC5788566

[139] Maude SL, Laetsch TW, Buechner J, Rives S, Boyer M, Bittencourt H, et al. Tisagenlecleucel in children and young adults with B-Cell lymphoblastic leukemia. N Engl J Med. 2018;378(5):439–48. 10.1056/NEJMoa1709866. 29385370 10.1056/NEJMoa1709866 PMC5996391

[140] San-Miguel J, Dhakal B, Yong K, Spencer A, Anguille S, Mateos MV, et al. Cilta-cel or standard care in Lenalidomide-Refractory multiple myeloma. N Engl J Med. 2023;389(4):335–47. 10.1056/NEJMoa2303379. 37272512 10.1056/NEJMoa2303379

[141] Munshi NC, Anderson LD Jr, Shah N, Madduri D, Berdeja J, Lonial S, et al. Idecabtagene vicleucel in relapsed and refractory multiple myeloma. N Engl J Med. 2021;384(8):705–16. 10.1056/NEJMoa2024850. 33626253 10.1056/NEJMoa2024850

[142] June CH, Sadelain M. Chimeric antigen receptor therapy. N Engl J Med. 2018;379(1):64–73. 10.1056/NEJMra1706169. 29972754 10.1056/NEJMra1706169 PMC7433347

[143] Newick K, O’Brien S, Moon E, Albelda SM. CAR t cell therapy for solid tumors. Annu Rev Med. 2017;68:139–52. 10.1146/annurev-med-062315-120245. 27860544 10.1146/annurev-med-062315-120245

[144] Wang JS, Schellenberg SJ, Demeros A, Lin AY. Exosomes in review: A new frontier in CAR-T cell therapies. Neoplasia. 2025;62:101147. 10.1016/j.neo.2025.101147. 40037165 10.1016/j.neo.2025.101147 PMC11923832

[145] Yang P, Cao X, Cai H, Feng P, Chen X, Zhu Y, et al. The exosomes derived from CAR-T cell efficiently target mesothelin and reduce triple-negative breast cancer growth. Cell Immunol. 2021;360:104262. 10.1016/j.cellimm.2020.104262. 33373818 10.1016/j.cellimm.2020.104262

[146] Sani F, Shojaei S, Tabatabaei SA, Khorraminejad-Shirazi M, Latifi M, Sani M, et al. CAR-T cell-derived exosomes: a new perspective for cancer therapy. Stem Cell Res Ther. 2024;15(1):174. 10.1186/s13287-024-03783-4. 38886844 10.1186/s13287-024-03783-4 PMC11184895

[147] Tang XJ, Sun XY, Huang KM, Zhang L, Yang ZS, Zou DD, et al. Therapeutic potential of CAR-T cell-derived exosomes: a cell-free modality for targeted cancer therapy. Oncotarget. 2015;6(42):44179–90. 10.18632/oncotarget.6175. 26496034 10.18632/oncotarget.6175 PMC4792550

[148] Zhu L, Kalimuthu S, Gangadaran P, Oh JM, Lee HW, Baek SH, et al. Exosomes derived from natural killer cells exert therapeutic effect in melanoma. Theranostics. 2017;7(10):2732–45. 10.7150/thno.18752. 28819459 10.7150/thno.18752 PMC5558565

[149] Spokeviciute B, Kholia S, Brizzi MF. Chimeric antigen receptor (CAR) T-cell therapy: harnessing extracellular vesicles for enhanced efficacy. Pharmacol Res. 2024;208:107352. 10.1016/j.phrs.2024.107352. 39147005 10.1016/j.phrs.2024.107352

[150] Zhao X, Zhao B, Sun Y, Liu A. CAR-exosomes derived from immune cells: an emerging nanoscale vanguard in overcoming tumor immunotherapy hurdles. Front Immunol. 2025;16:1655095. 10.3389/fimmu.2025.1655095. 40904456 10.3389/fimmu.2025.1655095 PMC12401991

[151] Lanuti P, Guardalupi F, Corradi G, Florio R, Brocco D, Veschi S, et al. CD19.CAR T-cell-derived extracellular vesicles express CAR and kill leukemic cells, contributing to antineoplastic therapy. Blood Adv. 2025;9(12):2907–19. 10.1182/bloodadvances.2024014860. 39903124 10.1182/bloodadvances.2024014860 PMC12180994

[152] Aharon A, Horn G, Bar-Lev TH, Zagagi Yohay E, Waks T, Levin M, et al. Extracellular vesicles derived from chimeric antigen receptor-T cells: a potential therapy for cancer. Hum Gene Ther. 2021;32:1224–41. 10.1089/hum.2021.192. 34494460 10.1089/hum.2021.192

[153] Zhang H, Li X, Pang H, Ma F, Hu A, Li Y, et al. CAR-T exosomes-platelet hybrid vesicles dual targeting against breast cancer recurrence and metastasis. Chem Eng J. 2025;525:169610. 10.1016/j.cej.2025.169610.

[154] Shimabukuro-Vornhagen A, Gödel P, Subklewe M, Stemmler HJ, Schlößer HA, Schlaak M, et al. Cytokine release syndrome. J Immunother Cancer. 2018;6(1):56. 10.1186/s40425-018-0343-9. 29907163 10.1186/s40425-018-0343-9 PMC6003181

[155] Lee DW, Gardner R, Porter DL, Louis CU, Ahmed N, Jensen M, et al. Current concepts in the diagnosis and management of cytokine release syndrome. Blood. 2014;2(2):188–95. 10.1182/blood-2014-05-552729. 10.1182/blood-2014-05-552729 PMC4093680 24876563

[156] Neelapu SS, Tummala S, Kebriaei P, Wierda W, Gutierrez C, Locke FL, et al. Chimeric antigen receptor T-cell therapy - assessment and management of toxicities. Nat Rev Clin Oncol. 2018;15(1):47–62. 10.1038/nrclinonc.2017. 28925994 10.1038/nrclinonc.2017.148 PMC6733403

[157] Calvo V, Izquierdo M. T lymphocyte and CAR-T cell-derived extracellular vesicles and their applications in cancer therapy. Cells. 2022;11(5):790. 10.3390/cells11050790. 35269412 10.3390/cells11050790 PMC8909086

[158] Anel A, Gallego-Lleyda A, de Miguel D, Naval J, Martínez-Lostao L. Role of exosomes in the regulation of T-cell mediated immune responses and in autoimmune disease. Cells. 2019;8(2):154. 10.3390/cells8020154. 30759880 10.3390/cells8020154 PMC6406439

[159] Li Q, Wang H, Peng H, Huyan T, Cacalano NA. Exosomes: versatile nano mediators of immune regulation. Cancers (Basel). 2019;11(10):1557. 10.3390/cancers11101557. 31615107 10.3390/cancers11101557 PMC6826959

[160] Hu D, Yang R, Wang G, Li H, Fan X, Liang G. Emerging strategies to overcome current CAR-T therapy dilemmas - exosomes derived from CAR-T cells. Int J Nanomed. 2024;19:2773–91. 10.2147/IJN.S445101. 10.2147/IJN.S445101 PMC10959326 38525009

[161] Lener T, Gimona M, Aigner L, Börger V, Buzas E, Camussi G, et al. Applying extracellular vesicles based therapeutics in clinical trials - an ISEV position paper. J Extracell Vesicles. 2015;4(1):30087. 10.3402/jev.v4.30087. 26725829 10.3402/jev.v4.30087 PMC4698466

[162] Andaloussi S, Mäger I, Breakefield XO, Wood MJ. Extracellular vesicles: biology and emerging therapeutic opportunities. Nat Rev Drug Discov. 2013;12(5):347–57. 10.1038/nrd3978. 23584393 10.1038/nrd3978

[163] Li J, Liu C, Zhang P, Shen L, Qi C. Optimizing CAR T cell therapy for solid tumours: a clinical perspective. Nat Rev Clin Oncol. 2025;22(12):953–68. 10.1038/s41571-025-01075-1. 41039013 10.1038/s41571-025-01075-1

[164] Saka OM, Dora DD, Kibar G, Tevlek A. Expanding the role of exosomes in drug, biomolecule, and nanoparticle delivery. Life Sci. 2025;368:123499. 10.1016/j.lfs.2025.123499. 39993468 10.1016/j.lfs.2025.123499

[165] Morse MA, Garst J, Osada T, Khan S, Hobeika A, Clay TM, et al. A phase I study of dexosome immunotherapy in patients with advanced non-small cell lung cancer. J Transl Med. 2005;3(1):9. 10.1186/1479-5876-3-9. 15723705 10.1186/1479-5876-3-9 PMC551593

[166] Buzas EI. The roles of extracellular vesicles in the immune system. Nat Rev Immunol. 2023;23(4):236–50. 10.1038/s41577-022-00763-8. 35927511 10.1038/s41577-022-00763-8 PMC9361922

[167] Sterner RC, Sterner RM. CAR-T cell therapy: current limitations and potential strategies. Blood Cancer J. 2021;11(4):69. 10.1038/s41408-021-00459-7. 33824268 10.1038/s41408-021-00459-7 PMC8024391

[168] Kamerkar S, LeBleu VS, Sugimoto H, Yang S, Ruivo CF, Melo SA, et al. Exosomes facilitate therapeutic targeting of oncogenic KRAS in pancreatic cancer. Nature. 2017;546(7659):498–503. 10.1038/nature22341. 28607485 10.1038/nature22341 PMC5538883

[169] Jang SC, Kim OY, Yoon CM, Choi DS, Roh TY, Park J, et al. Bioinspired exosome-mimetic nanovesicles for targeted delivery of chemotherapeutics to malignant tumors. ACS Nano. 2013;7(9):7698–710. 10.1021/nn402232g. 24004438 10.1021/nn402232g

[170] Ohno S, Takanashi M, Sudo K, Ueda S, Ishikawa A, Matsuyama N, et al. Systemically injected exosomes targeted to EGFR deliver antitumor microRNA to breast cancer cells. Mol Ther. 2013;21(1):185–91. 10.1038/mt.2012.180. 23032975 10.1038/mt.2012.180 PMC3538304

[171] Escrevente C, Keller S, Altevogt P, Costa J. Interaction and uptake of exosomes by ovarian cancer cells. BMC Cancer. 2011;11:108. 10.1186/1471-2407-11-108. 21439085 10.1186/1471-2407-11-108 PMC3072949

[172] Maia J, Caja S, Strano Moraes MC, Couto N, Costa-Silva B. Exosome-based cell-cell communication in the tumor microenvironment. Front Cell Dev Biol. 2018;6:18. 10.3389/fcell.2018.00018. 29515996 10.3389/fcell.2018.00018 PMC5826063

[173] Kar R, Dhar R, Mukherjee S, Nag S, Gorai S, Mukerjee N, et al. Exosome-based smart drug delivery tool for cancer theranostics. ACS Biomater Sci Eng. 2023;9(2):577–94. 10.1021/acsbiomaterials.2c01329. 36621949 10.1021/acsbiomaterials.2c01329 PMC9930096

[174] Barile L, Vassalli G. Exosomes: therapy delivery tools and biomarkers of diseases. Pharmacol Ther. 2017;174:63–78. 10.1016/j.pharmthera.2017.02.020. 28202367 10.1016/j.pharmthera.2017.02.020

[175] Kim MS, Haney MJ, Zhao Y, Mahajan V, Deygen I, Klyachko NL, et al. Development of exosome-encapsulated paclitaxel to overcome MDR in cancer cells. Nanomedicine. 2016;12(3):655–64. 10.1016/j.nano.2015.10.012. 26586551 10.1016/j.nano.2015.10.012 PMC4809755

[176] Zitvogel L, Regnault A, Lozier A, Wolfers J, Flament C, Tenza D, et al. Eradication of established murine tumors using a novel cell-free vaccine: dendritic cell-derived exosomes. Nat Med. 1998;4(5):594–600. 10.1038/nm0598-594. 9585234 10.1038/nm0598-594

[177] Wang R, Zhu T, Hou B, Huang X. An iPSC-derived exosome-pulsed dendritic cell vaccine boosts antitumor immunity in melanoma. Mol Ther. 2023;31(8):2376–90. 10.1016/j.ymthe.2023.06.005. 37312452 10.1016/j.ymthe.2023.06.005 PMC10422017

[178] Shubhra QTH, Veranič P, Wang Z. Amplifying antitumor immunity with iPSC-derived exosomes. Mol Ther. 2023;31(8):2300–1. 10.1016/j.ymthe.2023.07.001. 37467746 10.1016/j.ymthe.2023.07.001 PMC10422008

[179] Wu X, Meng Y, Yao Z, Lin X, Hu M, Cai S, et al. Extracellular vesicles as nature’s nano carriers in cancer therapy: insights toward preclinical studies and clinical applications. Pharmacol Res. 2025;217:107751. 10.1016/j.phrs.2025.107751. 40345354 10.1016/j.phrs.2025.107751

[180] In H, Park M, Lee H, Han KH. Immune cell engagers: advancing precision immunotherapy for cancer treatment. Antibodies. 2025;14(1):16. 10.3390/antib14010016. 39982231 10.3390/antib14010016 PMC11843982

[181] Abdalla AME, Xiao L, Miao Y, Huang L, Fadlallah GM, Gauthier M, et al. Nanotechnology promotes genetic and functional modifications of therapeutic T cells against cancer. Adv Sci. 2020;7(10):1903164. 10.1002/advs.201903164. 10.1002/advs.201903164 PMC7237845 32440473

[182] Yetisgin AA, Cetinel S, Zuvin M, Kosar A, Kutlu O. Therapeutic nanoparticles and their targeted delivery applications. Molecules. 2020;25(9):2193. 10.3390/molecules25092193. 32397080 10.3390/molecules25092193 PMC7248934

[183] Exosomes. Accessed September 12, – The Good, Bad, Ugly and Current State. Available online at: https://www.americanpharmaceuticalreview.com/Featured-Articles/575432-Exosomes-The-Good-Bad-Ugly-and-Current-State/ (2025).

[184] Zeng Y, Li S, Zhang S, Wang L, Yuan H, Hu F. Cell membrane coated-nanoparticles for cancer immunotherapy. Acta Pharm Sin B. 2022;12(8):3233–54. 10.1016/j.apsb.2022.02.023. 35967284 10.1016/j.apsb.2022.02.023 PMC9366230

[185] Kalluri VS, Smaglo BG, Mahadevan KK, Kirtley ML, McAndrews KM, Mendt M, et al. Engineered exosomes with KrasG12D specific siRNA in pancreatic cancer: a phase I study with immunological correlates. Nat Commun. 2025;16(1):8696. 10.1038/s41467-025-63718-2. 41027940 10.1038/s41467-025-63718-2 PMC12485160

[186] Yu W, Hurley J, Roberts D, Chakrabortty SK, Enderle D, Noerholm M, et al. Exosome-based liquid biopsies in cancer: opportunities and challenges. Ann Oncol. 2021;32(4):466–77. 10.1016/j.annonc.2021.01.074. 33548389 10.1016/j.annonc.2021.01.074 PMC8268076

[187] Johnson LR, Lee DY, Eacret JS, Ye D, June CH, Minn AJ. The immunostimulatory RNA RN7SL1 enables CAR-T cells to enhance autonomous and endogenous immune function. Cell. 2021;184(19):4981-4995.e14. 10.1016/j.cell.2021.08.004. 34464586 10.1016/j.cell.2021.08.004 PMC11338632

[188] Zhu T, Chen Z, Jiang G, Huang X. Sequential targeting hybrid nanovesicles composed of chimeric antigen receptor T-cell-derived exosomes and liposomes for enhanced cancer immunochemotherapy. ACS Nano. 2023;17(17):16770–86. 10.1021/acsnano.3c03456. 37624742 10.1021/acsnano.3c03456

[189] Soltanmohammadi F, Gharehbaba AM, Zangi AR, Adibkia K, Javadzadeh Y. Current knowledge of hybrid nanoplatforms composed of exosomes and organic/inorganic nanoparticles for disease treatment and cell/tissue imaging. Biomed Pharmacother. 2024;178:117248. 10.1016/j.biopha.2024.117248. 39098179 10.1016/j.biopha.2024.117248

[190] Yang Z, Zhong W, Yang L, Wen P, Luo Y, Wu C. The emerging role of exosomes in radiotherapy. Cell Commun Signal. 2022;20(1):171. 10.1186/s12964-022-00986-1. 36316715 10.1186/s12964-022-00986-1 PMC9620591

[191] Taghikhani A, Farzaneh F, Sharifzad F, Mardpour S, Ebrahimi M, Hassan ZM. Engineered tumor-derived extracellular vesicles: potentials in cancer immunotherapy. Front Immunol. 2020;11:221. 10.3389/fimmu.2020.00221. 32210954 10.3389/fimmu.2020.00221 PMC7069476

[192] Ye J, Li D, Jie Y, Luo H, Zhang W, Qiu C. Exosome-based nanoparticles and cancer immunotherapy. Biomed Pharmacother. 2024;179:117296. 10.1016/j.biopha.2024.117296. 39167842 10.1016/j.biopha.2024.117296

[193] Wang Z, Li P, Zeng X, Guo J, Zhang C, Fan Z, et al. CAR-T therapy dilemma and innovative design strategies for next generation. Cell Death Dis. 2025;16(1):211. 10.1038/s41419-025-07454-x. 40148310 10.1038/s41419-025-07454-x PMC11950394

[194] Lu M, Xing H, Zhao X, Huang Y, Zheng A, Liang XJ. Engineered extracellular vesicles as a next-generation vaccine platform. Matter. 2024;7(12):4180–205. 10.1016/j.matt.2024.09.012.

[195] Li Q, Xing H, Naeem A, Zhang K, Zheng A, Huang Y, et al. Extracellular vesicle-based mRNA therapeutics and vaccines. Exploration. 2025. 10.1002/EXP.20240109. 41476648 10.1002/EXP.20240109 PMC12752554

[196] Fang RH, Gao W, Zhang L. Targeting drugs to tumours using cell membrane-coated nanoparticles. Nat Rev Clin Oncol. 2023;20(1):33–48. 10.1038/s41571-022-00699-x. 36307534 10.1038/s41571-022-00699-x

[197] Li Y, Xu C, Lei C. The delivery and activation of growth factors using nanomaterials for bone repair. Pharmaceutics. 2023;15(3):1017. 10.3390/pharmaceutics15031017. 36986877 10.3390/pharmaceutics15031017 PMC10052849

[198] Danilushkina AA, Emene CC, Barlev NA, Gomzikova MO. Strategies for engineering of extracellular vesicles. Int J Mol Sci. 2023;24(17):13247. 10.3390/ijms241713247. 37686050 10.3390/ijms241713247 PMC10488046

[199] Moholkar DN, Kandimalla R, Gupta RC, Aqil F. Advances in lipid-based carriers for cancer therapeutics: liposomes, exosomes and hybrid exosomes. Cancer Lett. 2023;565:216220. 10.1016/j.canlet.2023.216220. 37209944 10.1016/j.canlet.2023.216220 PMC10325927

[200] Marquez CA, Oh CI, Ahn G, Shin WR, Kim YH, Ahn JY. Synergistic vesicle-vector systems for targeted delivery. J Nanobiotechnology. 2024;22(1):6. 10.1186/s12951-023-02275-6. 38167116 10.1186/s12951-023-02275-6 PMC10763086

[201] Maeki M, Tokeshi M. Engineered and artificial exosomes for Non-viral drug delivery nanocarriers. In: Baba Y, Hanayama R, Akita H, Yasui T, editors. Extracellular fine particles. Singapore: Springer; 2025. pp. 275–90. 10.1007/978-981-97-7067-0_19.

[202] Sato Y, Zhang W, Baba T, Chung U, Teramura Y. Extracellular vesicle-liposome hybrids via membrane fusion using cell-penetrating peptide-conjugated lipids. Regen Ther. 2024;26:533–40. 10.1016/j.reth.2024.07.006. 39165408 10.1016/j.reth.2024.07.006 PMC11333910

[203] Shiraishi K, Yokoyama M. Toxicity and immunogenicity concerns related to PEGylated-micelle carrier systems: a review. Sci Technol Adv Mater. 2019;20(1):324–36. 10.1080/14686996.2019.1590126. 31068982 10.1080/14686996.2019.1590126 PMC6493319

[204] Loukanov AR, Gagov H. High-resolution subunit detection of glutamate receptor by ultrasmall gold nanoparticles. Microsc Res Tech. 2012;75(9):1159–64. 10.1002/jemt.22043. 22461110 10.1002/jemt.22043

[205] Jia G, Han Y, An Y, Ding Y, He C, Wang X, et al. NRP-1 targeted and cargo-loaded exosomes facilitate simultaneous imaging and therapy of glioma in vitro and in vivo. Biomaterials. 2018;178:302–16. 10.1016/j.biomaterials.2018.06.029. 29982104 10.1016/j.biomaterials.2018.06.029

[206] Candas-Green D, Xie B, Huang J, Fan M, Wang A, Menaa C, et al. Dual blockade of CD47 and HER2 eliminates radioresistant breast cancer cells. Nat Commun. 2020;11(1):4591. 10.1038/s41467-020-18245-7. 32929084 10.1038/s41467-020-18245-7 PMC7490264

[207] Yu S, Jiang S, Zhou Y, Zhu Z, Yang X. Impact of radiation on exosomes in regulating tumor immune microenvironment. Adv Radiat Oncol. 2024;9(8):101549. 10.1016/j.adro.2024.101549. 39055959 10.1016/j.adro.2024.101549 PMC11269846

[208] Rzeszowska-Wolny J, Przybyszewski WM, Widel M. Ionizing radiation-induced bystander effects, potential targets for modulation of radiotherapy. Eur J Pharmacol. 2009;625(1–3):156–64. 10.1016/j.ejphar.2009.07.028. 19835860 10.1016/j.ejphar.2009.07.028

[209] Mannavola F, D’Oronzo S, Cives M, Stucci LS, Ranieri G, Silvestris F, et al. Extracellular vesicles and epigenetic modifications are hallmarks of melanoma progression. Int J Mol Sci. 2019;21(1):52. 10.3390/ijms21010052. 31861757 10.3390/ijms21010052 PMC6981648

[210] Huang CS, Ho JY, Chiang JH, Yu CP, Yu DS. Exosome-derived LINC00960 and LINC02470 promote the epithelial-mesenchymal transition and aggressiveness of bladder cancer cells. Cells. 2020;9(6):1419. 10.3390/cells9061419. 32517366 10.3390/cells9061419 PMC7349410

[211] Jassi C, Kuo WW, Kuo CH, Chang CM, Chen MC, Shih TC, et al. Mediation of radiation-induced bystander effect and epigenetic modification: the role of exosomes in cancer radioresistance. Heliyon. 2024;10(14):e34460. 39114003 10.1016/j.heliyon.2024.e34460 PMC11304029

[212] Umezu T, Tadokoro H, Azuma K, Yoshizawa S, Ohyashiki K, Ohyashiki JH. Exosomal miR-135b shed from hypoxic multiple myeloma cells enhances angiogenesis by targeting factor-inhibiting HIF-1. Blood. 2014;124(25):3748–57. 10.1182/blood-2014-05-576116. 25320245 10.1182/blood-2014-05-576116 PMC4263983

[213] Chen F, Xu B, Li J, Yang X, Gu J, Yao X, et al. Hypoxic tumour cell-derived exosomal miR-340-5p promotes radioresistance of oesophageal squamous cell carcinoma via KLF10. J Exp Clin Cancer Res. 2021;40(1):38. 10.1186/s13046-021-01834-9. 33485367 10.1186/s13046-021-01834-9 PMC7825246

[214] Rosenberg SA, Restifo NP, Yang JC, Morgan RA, Dudley ME. Adoptive cell transfer: a clinical path to effective cancer immunotherapy. Nat Rev Cancer. 2008;8(4):299–308. 10.1038/nrc2355. 18354418 10.1038/nrc2355 PMC2553205

[215] FDA. Approved Cellular and Gene Therapy Products. Available online at: https://www.fda.gov/vaccines-blood-biologics/cellular-gene-therapy-products/approved-cellular-and-gene-therapy-products (Accessed September 12, 2025).

[216] Mizenko RR, Feaver M, Bozkurt BT, Lowe N, Nguyen B, Huang KW, et al. A critical systematic review of extracellular vesicle clinical trials. J Extracell Vesicles. 2024;13(10):e12510. 10.1002/jev2.12510. 39330928 10.1002/jev2.12510 PMC11428870

[217] Riazifar M, Mohammadi MR, Pone EJ, Yeri A, Lässer C, Segaliny AI, et al. Stem cell-derived exosomes as nanotherapeutics for autoimmune and neurodegenerative disorders. ACS Nano. 2019;13(6):6670–88. 10.1021/acsnano.9b01004. 31117376 10.1021/acsnano.9b01004 PMC6880946

[218] Lamichhane TN, Sokic S, Schardt JS, Raiker RS, Lin JW, Jay SM. Emerging roles for extracellular vesicles in tissue engineering and regenerative medicine. Tissue Eng Part B Rev. 2015;21(1):45–54. 10.1089/ten.TEB.2014.0300. 24957510 10.1089/ten.teb.2014.0300 PMC4321981

[219] Ng CY, Kee LT, Al-Masawa ME, Lee QH, Subramaniam T, Kok D, et al. Scalable production of extracellular vesicles and its therapeutic values: a review. Int J Mol Sci. 2022;23(14):7986. 10.3390/ijms23147986. 35887332 10.3390/ijms23147986 PMC9315612

[220] FDA. Public Safety Notification on Exosome Products. Available online at: https://www.fda.gov/vaccines-blood-biologics/safety-availability-biologics/public-safety-notification-exosome-products (Accessed September 12, 2025).

[221] EMA. ATMP classification recommendations. Available online at: https://www.ema.europa.eu/en/human-regulatory/overview/advanced-therapy-medicinal-products-overview#classification-of-atmps-section (Accessed September 12, 2025).

[222] Tsuchiya A, Terai S, Horiguchi I, Homma Y, Saito A, Nakamura N, et al. Basic points to consider regarding the preparation of extracellular vesicles and their clinical applications in Japan. Regen Ther. 2022;21:19–24. 10.1016/j.reth.2022.05.003. 35619946 10.1016/j.reth.2022.05.003 PMC9127121

[223] Wang CK, Tsai TH, Lee CH. Regulation of exosomes as biologic medicines: regulatory challenges faced in exosome development and manufacturing processes. Clin Transl Sci. 2024;17(8):e13904. 10.1111/cts.13904. 39115257 10.1111/cts.13904 PMC11307316

[224] Pavlic J, Fury B, Mayo J, Herrera X, Jones S, Bergum D, et al. Optimizing CAR-T cell therapy: reducing manufacturing time and examining T cell memory phenotypes in B cell lymphoma. Blood. 2024;144(Suppl 1):7254. 10.1182/blood-2024-204047.

[225] Lin JK, Muffly LS, Spinner MA, Barnes JI, Owens DK, Goldhaber-Fiebert JD. Cost effectiveness of chimeric antigen receptor T-cell therapy in multiply relapsed or refractory adult large B-cell lymphoma. J Clin Oncol. 2019;37(24):2105–19. 10.1200/JCO.18.02079. 31157579 10.1200/JCO.18.02079

[226] Feng P, Zhang X, Gao J, Jiang L, Li Y. The roles of exosomes in anti-cancer drugs. Cancer Med. 2025;14(9):e70897. 10.1002/cam4.70897. 40298189 10.1002/cam4.70897 PMC12038748

[227] Li X, Corbett AL, Taatizadeh E, Tasnim N, Little JP, Garnis C, et al. Challenges and opportunities in exosome research-perspectives from biology, engineering, and cancer therapy. APL Bioeng. 2019;3(1):011503. 10.1063/1.5087122. 31069333 10.1063/1.5087122 PMC6481742

[228] Palakurthi SS, Shah B, Kapre S, Charbe N, Immanuel S, Pasham S, et al. A comprehensive review of challenges and advances in exosome-based drug delivery systems. Nanoscale Adv. 2024;6(23):5803–26. 10.1039/d4na00501e. 39484149 10.1039/d4na00501e PMC11523810

[229] Ranjan P, Colin K, Dutta RK, Verma SK. Challenges and future scope of exosomes in the treatment of cardiovascular diseases. J Physiol. 2023;601(22):4873–93. 10.1113/JP282053. 36398654 10.1113/JP282053 PMC10192497

[230] Sluijter JPG, Davidson SM, Boulanger CM, Buzás EI, de Kleijn DPV, Engel FB, et al. Extracellular vesicles in diagnostics and therapy of the ischaemic heart: position paper from the working group on cellular biology of the heart of the European Society of Cardiology. Cardiovasc Res. 2018;114(1):19–34. 10.1093/cvr/cvx211. 29106545 10.1093/cvr/cvx211 PMC5852624

[231] Liu JJJ, Liu D, To SKY, Wong AST. Exosomes in cancer nanomedicine: biotechnological advancements and innovations. Mol Cancer. 2025;24(1):166. 10.1186/s12943-025-02372-0. 40481526 10.1186/s12943-025-02372-0 PMC12144782

[232] Youssef E, Palmer D, Fletcher B, Vaughn R. Exosomes in precision oncology and beyond: from bench to bedside in diagnostics and therapeutics. Cancers (Basel). 2025;17(6):940. 10.3390/cancers17060940. 40149276 10.3390/cancers17060940 PMC11940788

[233] Huang S, Yan F, Qiu Y, Liu T, Zhang W, Yang Y, et al. Exosomes in inflammation and cancer: from bench to bedside applications. Mol Biomed. 2025;6(1):41. 10.1186/s43556-025-00280-9. 40490663 10.1186/s43556-025-00280-9 PMC12149089

[234] Picchio V, Pontecorvi V, Dhori X, Bordin A, Floris E, Cozzolino C, et al. The emerging role of artificial intelligence applied to exosome analysis: from cancer biology to other biomedical fields. Life Sci. 2025;375:123752. 10.1016/j.lfs.2025.123752. 40409585 10.1016/j.lfs.2025.123752

[235] Li J, Wang J, Chen Z. Emerging role of exosomes in cancer therapy: progress and challenges. Mol Cancer. 2025;24(1):13. 10.1186/s12943-024-02215-4. 39806451 10.1186/s12943-024-02215-4 PMC11727182

[236] Mendonca SR, Bangera PD, Keerikkadu M, Tippavajhala VK, Rathnanand M. Multifunctional engineering of exosomes for precision therapeutics: strategies for targeted delivery, barrier evasion, and clinical translation. Pharm Res. 2025. 10.1007/s11095-025-03961-w. 41188685 10.1007/s11095-025-03961-w PMC12698739

[237] Tandon R, Srivastava N. Unravelling exosome paradigm: therapeutic, diagnostic and theranostics application and regulatory consideration. Life Sci. 2025;366–367:123472. 10.1016/j.lfs.2025.123472. 39956185 10.1016/j.lfs.2025.123472

[238] Alsaidan OA. Current trends in exosomes as therapeutic drug delivery systems. Naunyn Schmiedebergs Arch Pharmacol. 2025 Oct;27. 10.1007/s00210-025-04615-9. 10.1007/s00210-025-04615-9 41143953

[239] Verma N, Arora S. Navigating the global regulatory landscape for exosome-based therapeutics: challenges, strategies, and future directions. Pharmaceutics. 2025;17(8):990. 10.3390/pharmaceutics17080990. 40871013 10.3390/pharmaceutics17080990 PMC12389065

[240] Song J, Song B, Yuan L, Yang G. Multiplexed strategies toward clinical translation of extracellular vesicles. Theranostics. 2022;12(15):6740–61. 10.7150/thno.75899. 36185609 10.7150/thno.75899 PMC9516239

[241] Jadhav S, Kumar A, Gulbake A. Exosomes: recent advances and challenges as targeted therapeutic delivery vesicles. Crit Rev Ther Drug Carrier Syst. 2023;40(4):101–33. 10.1615/CritRevTherDrugCarrierSyst.2022044495. 37075069 10.1615/CritRevTherDrugCarrierSyst.2022044495

[242] Li Y, Meng D, Cheng Y, Sun Y, Dong Y, Shi J, et al. Prospects of engineered exosomes in clinical applications: a review. Drug Dev Ind Pharm. 2025;51(11):1400–16. 10.1080/03639045.2025.2541789. 40736274 10.1080/03639045.2025.2541789

[243] Xu G, Jin J, Fu Z, Wang G, Lei X, Xu J, et al. Extracellular vesicle-based drug overview: research landscape, quality control and nonclinical evaluation strategies. Signal Transduct Target Ther. 2025;10(1):255. 40804047 10.1038/s41392-025-02312-w PMC12350758

[244] Eon-Duval A, Broly H, Gleixner R. Quality attributes of recombinant therapeutic proteins: an assessment of impact on safety and efficacy as part of a quality by design development approach. Biotechnol Prog. 2012(3). 10.1002/btpr.1548. 10.1002/btpr.1548 22473974

